# What Do Electronic Health Record Vendors Reveal About Their Products: An Analysis of Vendor Websites

**DOI:** 10.2196/jmir.2312

**Published:** 2013-02-19

**Authors:** Natalie K Yeung, Alejandro R Jadad, Aviv Shachak

**Affiliations:** ^1^University of TorontoFaculty of InformationToronto, ONCanada; ^2^University Health NetworkCentre for Global eHealth InnovationToronto, ONCanada; ^3^University Health NetworkElectronic Living Lab for Interdisciplinary Cancer Survivorship Research (Centre for Health, Wellness and Cancer Survivorship)Toronto, ONCanada; ^4^University of TorontoInstitute of Health Policy, Management and EvaluationToronto, ONCanada; ^5^University of TorontoDalla Lana School of Public HealthToronto, ONCanada; ^6^University of TorontoDepartment of AnesthesiaToronto, ONCanada

**Keywords:** Electronic health record, Vendors, Diffusion of Innovations, Websites

## Abstract

**Background:**

Purchasing electronic health records (EHRs) typically follows a process in which potential adopters actively seek information, compare alternatives, and form attitudes towards the product. A potential source of information on EHRs that can be used in the process is vendor websites. It is unclear how much product information is presented on EHR vendor websites or the extent of its value during EHR purchasing decisions.

**Objective:**

To explore what features of EHR systems are presented by vendors in Ontario, Canada, on their websites, and the persuasive means they use to market such systems; to compare the online information available about primary care EHR systems with that about hospital EHR systems, and with data compiled by OntarioMD, a regional certifying agency.

**Methods:**

A list of EHR systems available in Ontario was created. The contents of vendor websites were analyzed. A template for data collection and organization was developed and used to collect and organize information on the vendor, website content, and EHR features. First, we mapped information on system features to categories based on a framework from the Institute of Medicine (IOM). Second, we used a grounded theory–like approach to explore information for building consumer confidence in the vendor and product, and the various persuasive strategies employed on vendor websites. All data were first coded by one researcher. A peer reviewer independently analyzed a randomly chosen subset of the websites (10 of 21; 48%) and provided feedback towards a unified coding scheme. All data were then re-coded and categorized into themes. Finally, we compared information from vendor websites and data gathered by OntarioMD.

**Results:**

Vendors provided little specific product information on their websites. Only two of five acute care EHR websites (40%) and nine of 16 websites for primary care systems (56%) featured seven or all eight of the IOM components. Several vendor websites included system interface demonstrations: screenshots (six websites), public videos or slideshows (four websites), or for registered viewers only (three websites). Persuasive means used by vendors included testimonials on 14/21 (67%) websites, and directional language. Except for one free system, trial EHR versions were not available. OntarioMD provided more comprehensive information about primary care systems than the vendors’ websites. Of 14 points of comparison, only the inclusion of templates and bilingual interfaces were fully represented in both data sources. For all other categories, the vendor websites were less complete than the OntarioMD site.

**Conclusions:**

EHR vendor websites employ various persuasive means, but lack product-specific information and do not provide options for trying systems on a limited basis. This may impede the ability of potential adopters to form perceptions and compare various offerings. Both vendors and clients could benefit from greater transparency and more specific product information on the Web.

**Trial Registration:**

N/A

## Introduction

Purchasing electronic health record (EHR) systems is a process in which potential buyers and users often seek and assess information about the products in question and compare alternatives. EHR is often a new technology to the people who use it, introducing new ways of performing clinical and administrative tasks. As such, it may be regarded as an innovation. Rogers’ [1] diffusion of innovations theory suggests that the process of adopting innovations (the innovation decision process) typically follows five stages: knowledge, persuasion, decision, implementation, and confirmation. Most relevant to this work is the *knowledge* stage in which adopters learn about the existence of an innovation (awareness knowledge), gain basic knowledge of how to use it (how-to knowledge), and understand the underlying principles behind it (principles knowledge). This is followed by the *persuasion* stage, in which potential adopters actively seek more information about the innovation, evaluate its characteristics, form positive or negative attitudes toward it, and subsequently adopt (eg, purchase) or reject the innovation at the *decision* stage.

For EHRs, the adoption decision process involves a planning phase that includes needs assessment, identifying champions, gaining buy-in from stakeholders, workflow analysis, understanding financial issues, and goal setting [2,3]. This is followed by a system selection phase in which information is sought from various sources including vendors and general consultants[4], visits to practices that have installed systems of interest, and product demonstrations [2,3]. At this stage, according to Lorenzi et al [3], “the internet provides a valuable source of information regarding specific EHR system products, capabilities, and the selection process” (p.8). In particular, vendor websites could play an important role in making an adoption decision by creating awareness, providing how-to and principle knowledge, and using various persuasive means to affect potential adopters’ perceptions of EHRs. However, to the best of our knowledge, no systematic efforts have been made to examine whether EHR vendors use their websites to present the information typically gathered in the pre-decision stages of Rogers’ innovation-decision process.

To contribute to filling this gap, we studied the information provided on websites of EHR vendors operating in Ontario, Canada. The term EHR is used here broadly to encompass computerized systems containing patient information for direct clinical use. For simplicity, we use the term for both stand-alone electronic medical records (EMRs), in which information “can be created, gathered, managed, and consulted by authorized clinicians and staff within one health care organization” [5] (p. 6), and interoperable EHR systems, which may be operated by clinicians and staff across various health care organizations. The following research questions were investigated:

RQ1: What and how much product-specific information do Ontario EHR vendors reveal on their own websites or on external websites?

First, we examined what vendors reveal about the functional characteristics of their EHR products by looking for product-specific information related to eight core EHR functionalities defined by the Institute of Medicine (IOM) [6]. As a subset of this question, we explored what similarities and differences in this product-specific information exist between acute care (hospital-based) and primary care (family practice) EHR vendor websites.

Second, we compared the product-specific information presented on vendor websites with information presented on an external website. The selected external website is operated by a provincial agency (OntarioMD), which assists physicians in the transition from paper to electronic records and acts as a certifying body for primary care EHRs in Ontario [7].

RQ2: In what ways do Ontario EHR vendor websites attempt to persuade users to purchase their products?

For this purpose we looked at what persuasive means are used by vendors on their websites and considered how they could influence potential adopters’ perceptions of the systems. As a subset of this question, we also explored what differences in the persuasive means employed exist between acute care (hospital-based) and primary care (family practice) EHR vendor websites.

## Methods

### Vendor Website Selection

**Figure 1:**
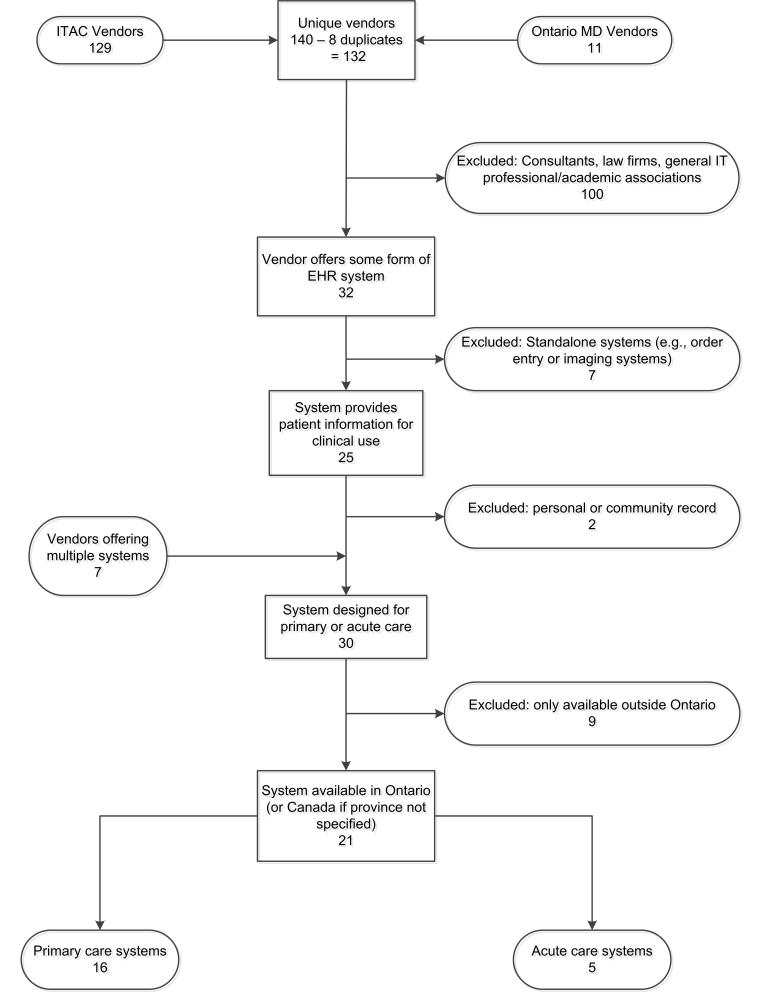
A flow chart of the vendor website selection process and results.

Figure 1 presents the website selection process. We compiled a list of EHR vendor websites for systems available in the province of Ontario, Canada, from two sources. The first is OntarioMD [7] (11 vendors), a provincial agency that works closely with physicians to provide support for the transition from paper to electronic records. It certifies primary care EHRs, so that physicians adopting them are eligible for funding from the province of Ontario under the Physician IT Program [8]. OntarioMD also publishes vendor responses to a number of standard questions on their website. The second is the Information Technology Association of Canada (ITAC) Health members list [9] (129 vendors), which was used to identify acute care EHR vendors and additional non-certified primary care vendors. This list contains contact information for information technology organizations that are active in the health care sector. ITAC Health, formerly known as the Canadian Healthcare Information Technology Trade Association (CHITTA) [10], is an established national industry association and so was considered a reputable, consistent, and reasonably comprehensive source of information.

After removing duplicates (8 vendors), systems were included based on: 1) specific mention of being or description of a product that could correspond to an EHR in the broad sense described above, 2) being designed and marketed for either primary or acute health care organizations but not for patients, 3) maintaining patient profiles and documentation for direct clinical use, and 4) availability and implementation in Ontario. These criteria excluded consultants, law firms, general IT, professional, and academic associations (100 websites). Specialized software such as computerized provider order entry systems not integrated within an EHR suite and picture archiving and communication systems (7 systems), personal or community health records (2 systems), and systems not available in Ontario (9 systems) were also excluded. Seven vendors offered multiple systems; therefore, the final list includes 21 systems (16 primary care and 5 acute care EHRs).

### Data Collection and Analysis

**Figure 2:**
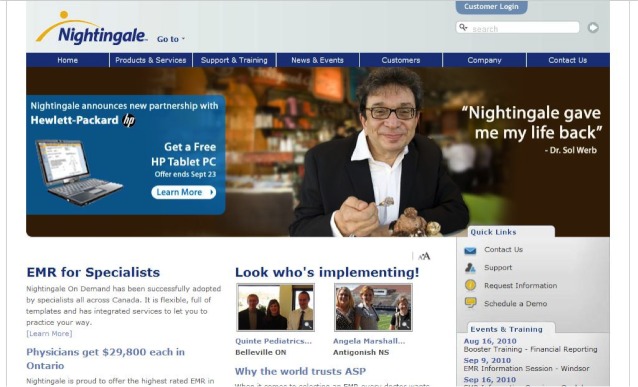
Nightingale On Demand (primary care). From: http://www.nightingalemd.ca; taken on August 25, 2010.

Each vendor website was examined for a number of general and system-specific characteristics. No pre-existing methodology was found for evaluating and classifying discrete website content elements independent of external accuracy or credibility. Therefore, an interpretive approach to data collection and analysis was employed. Based on a preliminary review of websites, as well as information from the literature, a template for data collection and organization was developed (Multimedia Appendix 1) and used to collect and organize information on the vendor, website content, and EHR system features. As described in detail below, product-specific and persuasive features were analyzed using framework analysis [11] and grounded theory-like [12,13] approaches, respectively. Finally, we compared the information presented on vendor websites with the information from OntarioMD website. Multimedia Appendix 2 and Multimedia Appendix 3 illustrate screen captures from vendor websites, and Figure 2 presents an example homepage.

#### Information About System Features

The first analysis addressed information related to EHR system features and specifications across both primary and acute care systems. This information was compiled from website texts (paragraphs or feature lists) and nontextual features, such as screenshots or other graphics. Data were drawn both from vendor websites and the OntarioMD site. Several possible frameworks for analyzing product-specific characteristic were examined including EHR component models and frameworks from HIMSS Analytics [14], Gartner Inc. [15], and IOM [6]. The IOM framework was selected for being the most detailed, comprehensive, and from an internationally reputable organization. Taking a framework analysis approach [11], data from websites were mapped to the eight core functionalities of an EHR as defined by the IOM [6]:

1. health information and data;
2. results management (eg, images, clinical dashboard, alerts);
3. order entry and management (eg, computerized provider order entry, prescribing);
4. decision support (eg, drug interactions, prevention and detection alerts);
5. electronic communication and connectivity (eg, email, integrated records);
6. patient support (eg, patient education content);
7. administrative processes (eg, patient scheduling, billing);
8. reporting and population health management (eg, quality indicators, national registries).

Each EHR system’s website was evaluated for a description of at least one feature in each of the eight EHR functionalities (or components) defined by the IOM. Features not explicitly mentioned were not considered present in the analysis. For instance, a website not stating that patient data were stored or displayed by the system would not meet the first functionality, although it would be reasonable to assume that the system must contain some patient data in order for any other functions to operate.

#### Analysis of Persuasive Features

Since a suitable analytic framework for evaluating persuasive features was not found, a grounded theory–like approach was employed to develop the themes on the basis of content extracted throughout the study from all of the websites included in this analysis. The first part of this evaluation focused on information presented to build *consumer confidence* in the vendors, their websites, and by extension, their products. The second part of the analysis focused on the *direct persuasive strategies* employed. We followed a typical iterative process of open coding, consensus building, re-coding, and category/theme development as described below. This strategy highlighted trends in vendor website information content and delivery and facilitated an investigation of the differences between primary and acute care vendor websites.

First, open coding [12] was employed. To ensure trustworthiness, a peer reviewer independently analyzed a randomly chosen subset of the websites (10 of 21; 48%) and provided feedback towards a unified coding scheme. All data were then re-coded and categorized into broad recurring themes that emerged. Category-building was also influenced by constructs designed by other researchers [16,17]. Finally, findings related to each of the categories were summarized for all vendor websites.

#### Comparison of Vendor Websites and OntarioMD Information

A comparison of online information sources about EHR systems was performed for primary care systems certified by OntarioMD. Systems with multiple certified versions were considered a single entity for comparison. Based on the OntarioMD information, 14 data points for each certified system were collected, and each point was checked to see if it also appeared on the corresponding vendor’s website.

## Results

A list of 120 ITAC Health members and 12 OntarioMD-certified systems was compiled in August 2010. From this list, 21 websites representing systems from 19 different vendors met the inclusion criteria. Table 1 presents a list of vendors and systems and their respective websites. Of these websites, 5 (24%) were for acute care systems and 16 (76%) for primary care systems.

**Table 1:** List of all systems included for analysis.

Vendor name	Product name (with version if available)	Vendor homepage (as of August 2010)	Archived homepage (13 Feb 2013)
**Primary care EHR** ^**a**^ **systems**			
ABELSoft Corp.	ABELMed EHR-EMR/PM v11 ^b^	http://www.abelsoft.com/	http://www.webcitation.org/6EPAunGBO
Alphaglobal-IT	Globe Med v1.0 ^b^	http://www.alpha-it.com	http://www.webcitation.org/6EP8OzEd7
Alphaglobal-IT	Universal eHealth MD (UHM) v5.0 ^b^	http://www.alpha-it.com	
CLINICARE Corp.	EliteCare v6.7 ^b^	http://www.clinicare.com/	http://www.webcitation.org/6EP8eZ5t1
Eclipsys Corp.	Sunrise Ambulatory Care	http://www.eclipsys.com/	No longer available
EMIS Inc.	EMIS system	http://www.emis.ca/	No longer available
GE Healthcare	Centricity	http://www.gehealthcare.com/	http://www.webcitation.org/6EP8tnrNO
Healthscreen Solutions Inc.	HS Practive v4.0 ^b^	http://www.healthscreen.com/	http://www.webcitation.org/6EP8wsiyc
Jonoke Software Development Inc.	JonokeMed 5.1^b^	http://www.jonoke.com/	http://www.webcitation.org/6EP90N0Mh
(McMaster University, Department of Family Medicine)	OSCAR v9.06 (sometimes known as OSCAR McMaster) ^b^	http://oscarcanada.org/ or http://oscarmcmaster.org/	http://www.webcitation.org/6EP95PrX5
MD Physician Services Software Inc.	PS Suite v5.1 ^b^	http://www.practicesolutions.ca/index.cfm/ci_id/47452/la_id/1.htm	http://www.webcitation.org/6EP97aVjl
Nightingale Informatix Corp.	Nightingale On-Demand v8.3 ^b^	http://www.nightingalemd.ca/	http://www.webcitation.org/6EP9AE9V5
Optimed Software Corp.	Accuro	http://www.optimedsoftware.com/index.php	http://www.webcitation.org/6EP9CAzpP
P & P Data Systems Inc.	Clinic Information System (Clinic/Enterprise/Practice Editions, v.7.4.5) ^b^	http://www.p-pdata.com/	http://www.webcitation.org/6EP9ErrmK
xwave	Bell Aliant xwaveEMR v8 ^b^	http://www.xwave.com/	http://www.webcitation.org/6EP9GYkai
York-Med Systems Inc.	York-Med MD Suite v8.6 ^b^	http://www.york-med.com/	http://www.webcitation.org/6EP9Jhe3k
**Acute care EHR systems**			
B Sharp Technologies Inc.	B Care	http://www.bsharp.com/	http://www.webcitation.org/6EP9LSAke
Cerner Corp.	PowerChart EMR	http://www.cerner.com/public/default.asp?id=18731	No longer available
Eclipsys Corp.	Sunrise Clinical Manager	http://www.eclipsys.com/	No longer available
QuadraMed Corp.	QCPR	http://www.quadramed.com/	http://www.webcitation.org/6EP9RGAzO
TELUS Health Solutions	Oacis	http://telushealth.com/en/default.aspx	http://www.webcitation.org/6EP9UrNsc

^a^ Most of the primary care systems are in fact EMRs, but for simplicity we decided to use a single term (EHR) throughout.

^b^ System certified by OntarioMD.

### Information About System Features

In general, vendors provided little specific product information on their websites, and this was more pronounced for acute care vendor websites than primary care sites. As described in Table 2, none of the five acute care systems websites presented all eight IOM system components. On their respective websites, QuadraMed’s QCPR noted seven functionalities, while Cerner’s PowerChart mentioned only two. Furthermore, no single component was seen in all five systems. The importance of communication between providers was emphasized, with four system websites noting some form of secure electronic mail or messaging, as well as the ability to order tests or receive results electronically. Clinical decision support, such as drug-drug interactions, was also claimed on four of the five websites.

In contrast, many of the websites for primary care systems featured seven or all eight of the IOM components. ABELMed, JonokeMed, OSCAR, Practice Solutions PS Suite, and xwaveEMR described all functionalities, while another four systems were missing only one component. All 16 primary care systems met the definitions for the health information and administration functionalities. Primary care systems generally presented more components than acute care systems. The least commonly found component on websites of both types of systems was information on patient education features, which generally consisted of handouts and reference materials to be given to patients, outlining the details of relevant conditions, diagnoses, and treatment plans.

**Table 2:** Core functionalities of EHRs presented on websites (Y=website contains the feature).

Vendor (System)	Health info^a^	Results^b^	Order entry^c^	Decision support^d^	Connect^e^	Patient support^f^	Admin^g^	Reports^h^	Total
**Primary care EHR systems**									
ABELSoft (ABELMed)	Y	Y	Y	Y	Y	Y	Y	Y	8
AlphaIT (GlobeMed)	Y	Y	Y	Y	Y	Y	Y	–	7
AlphaIT (UHM)	Y	Y	Y	Y	Y	Y	Y	–	7
Clinicare (EliteCare)	Y	Y	Y	–	Y	–	Y	–	5
Eclipsys (Sunrise Ambulatory Care)	Y	–	Y	Y	Y	–	Y	–	5
EMIS	Y	Y	Y	Y	Y	–	Y	–	6
GE (Centricity)	Y	–	Y	Y	–	–	Y	Y	5
Healthscreen (HS Practice)	Y	–	–	–	–	–	Y	Y	3
Jonoke (JonokeMed)	Y	Y	Y	Y	Y	Y	Y	Y	8
Nightingale (On-Demand)	Y	–	Y	Y	Y	–	Y	Y	6
Optimed (Accuro)	Y	Y	Y	–	Y	–	Y	–	5
OSCAR	Y	Y	Y	Y	Y	Y	Y	Y	8
P&P Data Systems (CIS)	Y	Y	Y	Y	Y	–	Y	Y	7
Practice Solutions (PS Suite)	Y	Y	Y	Y	Y	Y	Y	Y	8
xwave (xwaveEMR)	Y	Y	Y	Y	Y	Y	Y	Y	8
York-Med (MD Suite)	Y	Y	Y	Y	Y	–	Y	Y	7
**Acute care EHR systems**									
B Sharp (B Care)	Y	–	–	–	Y	–	–	Y	3
Cerner (PowerChart)	–	–	Y	Y	–	–	–	–	2
Eclipsys (Sunrise Clinical Manager)	–	Y	Y	Y	Y	–	–	Y	5
QuadraMed (QCPR)	Y	Y	Y	Y	Y	Y	Y	–	7
Telus (oacis)	Y	Y	Y	Y	Y	–	–	Y	6
	19	15	19	17	18	8	17	13	

^a^ Health information & data

^b^ Results management

^c^ Order entry/management

^d^ Decision support

^e^ Electronic communication & connectivity

^f^ Patient support

^g^ Administrative processes

^h^ Reporting & population health management

### Persuasive Features

The main findings related to building consumer confidence and use of direct persuasive strategies for both acute and primary care vendor websites are discussed in detail below.

#### Information Building Consumer Confidence

The following aspects related to building consumer confidence in the vendors, their websites, and by extension, their products were identified: (1) last date of update; (2) external connections (ie, affiliations with or certification by associations, partners, and suppliers); and (3) customer support (eg, documentation, technical support, contact information). Findings related to each of these aspects are summarized in Table 3. First, the majority of sites were updated in 2010, the year in which data collection took place (11 of 16 (69%) primary care vendors, and 3 of 5 (60%) acute care vendors). Of the seven remaining sites, five were updated in 2008 or 2009, one was updated in 2007, and one had no update information.

**Table 3:** Means for establishing consumer confidence presented on vendor websites.

Vendor (System)	Last update	Affiliates	Client support ^m^
**Primary care EHR systems**			
ABELSoft (ABELMed)	2010	ONMD ^a^, MS ^b^	24/7 helpdesk
AlphaIT (GlobeMed)	2010	–	Client section ^n^
AlphaIT (UHM)	2010	–	Client section ^n^
Clinicare (EliteCare)	2008	IBM	Support section
Eclipsys (Sunrise Ambulatory Care)	2010	–	Standard only
EMIS	2010	MS ^b^, HP ^c^	Standard only
GE (Centricity)	2010	–	Customer portal ^n^
Healthscreen (HS Practice)	2008	OMA ^d^, COFP ^e^	Physician section ^n^
Jonoke (JonokeMed)	2009	BBB ^f^, Apple, CHITTA ^g^, Dell, LaCie	Client section ^n^
Nightingale (On-Demand)	2010	ONMD ^a^	Client section ^n^, unlimited tech support, documentation
Optimed (Accuro)	2010	Clinicare	Standard only
OSCAR	2010	ONMD ^a^, McMaster	Not standard; user society, listservs, blog
P&P Data Systems (CIS)	2010	ONMD, Dell, MS ^b^, HP ^c^, Sun	Client section ^n^, remote desktop
Practice Solutions (PS Suite)	2010	CMA ^h^, ONMD ^a^	Client portal ^n^
xwave (xwaveEMR)	2009	Bell Aliant, GE ^i^, ONMD ^a^, ITAC Health	Client section ^n^, helpdesk
York-Med (MD Suite)	2007	ONMD ^a^	Helpdesk, webcasts
**Acute care EHR systems**			
B Sharp (B Care)	2009	MS ^b^, Sun, client list	Standard only
Cerner (PowerChart)	2010	CCHIT ^j^	Standard only
Eclipsys (Sunrise Clinical Manager)	2010	–	Standard only
QuadraMed (QCPR)	n/a	HIMSS ^k^, AHIMA ^l^	Client section ^n^
Telus (oacis)	2010	–	Only phone, email

^a^ OntarioMD

^b^ Microsoft

^c^ Hewlett-Packard

^d^ Ontario Medical Association

^e^ College of Ontario Family Physicians

^f^ Better Business Bureau

^g^ Now ITAC Health

^h^ Canadian Medical Association

^i^ General Electric

^j^ Certification Commission for Health Information Technology

^k^ Healthcare Information and Management Systems Society

^l^ American Health Information Management Association

^m^ Client support includes standard contact information (phone, email, mailing address, optional fax) unless otherwise stated.

^n^ Viewer must be logged in.

Second, most vendors noted affiliations with technology companies or health-related associations on their websites. Six vendors did not have any affiliates or partners listed. Of the three acute care vendors who mentioned other organizations, Cerner and QuadraMed listed American health care associations (the Certification Commission for Healthcare Information Technology and the American Health Information Management Association, respectively). The third vendor, B Sharp, listed affiliations with technology companies such as Microsoft and Sun and displayed a client list of Ontario health care organizations.

Eight of the twelve primary care vendors with systems certified by OntarioMD (67%) mentioned this certification. However, this certification was not emphasized by most vendors and was separated from information about the system or mentioned only as a news item; only ABELMed conspicuously displayed the OntarioMD logo on its homepage. Seven primary care vendors listed affiliations with technology companies such as Dell and Microsoft. A number of health organizations were seen as well, such as the Ontario Medical Association (Healthscreen) and CHITTA/ITAC Health (Jonoke and xwave). Additionally, the Canadian Medical Association and McMaster University are unique in that they are the parent organizations of Practice Solutions and OSCAR, respectively.

All vendors but two provided “standard” contact information on their websites (phone number, online contact, mailing address, and possibly a fax number). The two that did not were OSCAR, which does not have a central location or head office, and Telus, which provided only a phone number and email address. OSCAR did not provide conventional contact information, but as an open source project there are listservs and blogs providing online support. Free membership in the OSCAR User Society was also encouraged to connect with other users, and the software source code is freely available. Third-party service providers support OSCAR implementations on a paid basis [18].

Of the acute care vendors, only QuadraMed went beyond the standard information to include a client-only section. In contrast, a number of primary care vendors mentioned providing 24/7 support in the form of phone lines or online help. One vendor (York-Med) advertised regular continuing education webcasts for clients. Thirteen of the 16 vendors (81%) provided some sort of client-only section on their website, presumably containing documentation and resources.

In order to give potential customers a better idea of their product, some vendors provided a demonstration of their system interface. Six websites posted only screenshots, three posted videos that were only available to viewers who logged into the site, and four had publicly available video demos. Of these four (AlphaIT UHM, OSCAR, Practice Solutions PS Suite, and xwaveEMR), only AlphaIT UHM and OSCAR went beyond a slideshow format and showed the system in active use. The full version of OSCAR can also be freely downloaded.

#### Direct Persuasive Strategies

The main categories of direct persuasive strategies used by vendors that emerged from the data were: (1) directional text (ie, text that encourages the user to identify with the system through the use of possessives, such as “your organization” or “your patients”); (2) customer testimonials; (3) online product demonstrations; and (4) topics addressed (general discussion topics around EHRs such as privacy and security concerns, cost savings or return on investment, and digitization of existing records). Table 4 provides an overview of vendors’ use of these direct persuasive strategies. Most vendors had some form of testimonial on their website. Seven of the 21 systems (33%) did not have testimonials, but three of those had space set aside for future testimonials. Of the 14 systems with testimonials, 12 were for primary care systems. Only two of the acute care vendors had testimonials (B Sharp and Telus), and one of these was on a PDF brochure instead of on the webpage itself. The most common form of testimonial was a short quote, often with part or all of the user’s name and organization. Some vendors extended the testimonials into case studies, going more in-depth into the client’s practice and implementation. EMIS and Telus each used a video testimonial instead of text.

**Table 4:** Direct persuasive strategies employed on vendor websites ^a^.

	Client Testimonials				Product Demos			Topics Discussed				
Vendor (System)	Blank Page	Short Text	Long Text	Video	Image	Video	Mock Site	1 ^b^	2 ^c^	3 ^d^	4 ^e^	Total
**Primary care EHR systems**												
ABELSoft (ABELMed)	–	Y	–	–	–	–	–	Y	–	Y	–	3
AlphaIT (GlobeMed)	Y	–	–	–	–	O	–	–	–	–	–	2
AlphaIT (UHM)	Y	–	–	–	–	Y	–	–	–	–	–	2
Clinicare (EliteCare)	–	Y	–	–	Y	–	–	–	–	–	–	2
Eclipsys (Sunrise Ambulatory Care)	–	–	–	–	–	–	–	Y	Y	–	–	2
EMIS	–	Y	–	Y	–	–	–	Y	–	Y	–	4
GE (Centricity)	–	–	–	O	–	O	–	Y	–	Y	–	4
Healthscreen (HS Practice)	–	Y	–	–	–	–	–	Y	–	Y	–	2
Jonoke (JonokeMed)	–	Y	–	–	Y	–	–	Y	Y	Y	–	5
Nightingale (On-Demand)	–	–	Y	–	–	O	–	–	–	–	–	2
Optimed (Accuro)	–	Y	–	–	Y	–	–	Y	–	–	Y	4
OSCAR	–	–	Y	–	Y	Y	O	–	–	Y	–	5
P&P Data Systems (CIS)	–	Y	–	–	Y	–	–	–	Y	–	Y	4
Practice Solutions (PS Suite)	–	–	Y	–	–	Y	–	–	Y	Y	Y	5
xwave (xwaveEMR)	–	–	Y	–	–	Y	–	–	Y	Y	Y	5
York-Med (MD Suite)	–	Y	–	–	–	–	–	–	–	Y	–	2
**Acute care EHR systems**												
B Sharp (B Care)	–	Y	–	–	–	–	–	–	Y	–	–	2
Cerner (PowerChart)	–	–	–	–	–	–	–	–	Y	–	–	1
Eclipsys (Sunrise Clinical Manager)	–	–	–	–	–	–	–	Y	Y	–	Y	3
QuadraMed (QCPR)	Y	–	–	–	–	–	–	Y	Y	–	Y	4
Telus (oacis)	–	–	–	Y	Y	–	–	Y	Y	Y	–	5
**Total**	3	9	4	3	6	7	1	10	10	10	6	

^a^ Labels: Y: Website contains feature; O: Website contains feature, but viewer must be logged in; –: Website does not contain feature.

^b^ 1: Quality of care

^c^ 2: Integration/interoperability

^d^ 3: Costs/Return on investment

^e^ 4: Practice efficiency/productivity

The use of text speaking directly to the intended audience was prevalent across vendor websites, with the single exception of the QuadraMed site. Other sites discussed their systems in relation to “your practice” and “your organization”. Some primary care vendors described features from a clinician’s point of view, using statements such as “you can easily draw pathology” (ABELMed) or “you [can] add sketches or pictures to a record” (JonokeMed). This directional text, combined with the use of testimonials, made it clear what audience each site intended to reach. The acute care vendors directed their sites to health organization executives and administration, or the people within the organization responsible for selecting and purchasing institutional software. In contrast, primary care sites were aimed very directly at physicians who owned their own practices or were part of a small group practice.

All of the vendors, except for Clinicare, went beyond system-specific issues and included general discussions surrounding the adoption of EHRs and implications for practice. Topics discussed by acute care EHR vendors included integration of data within an organization or with external organizations, and the resulting improvements in quality of care, resource efficiency or productivity. Primary care EHR vendor websites discussed the impact of EHR adoption on practice administration and workflow. Related to this, a number of vendors emphasized the training and support they provide to ease the transition and ensure that physicians are able to use EHRs comfortably. Several vendors stated that paper charts could be scanned into the system. Finally, nine of the primary care EHR vendors (56%) discussed the potential cost savings and return on investment. While none of the vendors quoted a price for their product (except for OSCAR, which is free), many stated that their systems would help offset costs through efficient billing and administration, eg, “Fee for Service physicians…will see an immediate improvement in cash flow, which can lead to significant savings to the practice over time.” (ABELMed Inc).

### Comparison of Vendor Websites and OntarioMD Information

The overlap between the information on vendor and OntarioMD websites for the 12 certified primary care systems is presented in Table 5. Vendor websites only contained a subset of the data gathered from the OntarioMD site; none of the vendors included all of their OntarioMD information on their own website. Of the 14 points of comparison between the OntarioMD data and the vendor websites, only the inclusion of templates and bilingual interfaces are fully represented in both data sources. For all of the other categories, the vendor websites were less complete than the OntarioMD site. None of the data missing from the OntarioMD information were subsequently found on vendor websites, and the vendor websites often did not go into as much detail as OntarioMD did. This was particularly noticeable for technical configuration specifications, which were broken down into optimal implementations for three specific scenarios in OntarioMD. Sites that provided configuration details, such as OSCAR and ABELMed, tended to provide general guidelines regarding compatible equipment and leave details such as the number of computers required to the discretion of the practice. Notably, a number of categories from OntarioMD website are not truly applicable to OSCAR, which is an open source system. Although it lists McMaster University as its vendor in OntarioMD, this is not the same vendor-product relationship as other systems, since third-party providers would handle services such as remote server management and backup.

**Table 5:** Information overlap between OntarioMD and vendor websites.

	Source (Ontario MD or Vendor)		
System Information	OntarioMD only	Both	Neither
Size of User Base	4	6	2
Training Program	4	8	0
Support Program	3	9	0
Frequency of System Upgrades	8	4	0
Conversion of Electronic Data	6	6	0
Health Card Validation	5	7	0
Data Entry Templates	0	12	0
Bilingual Interface	0	2	10
Clinical Coding Systems	2	10	0
Configuration Specifications	8	4	0
Remote Server Management	6	6	0
Member of a Vendor Collaborative Network	2	1	9
Health Canada Medical Device Licensing	1	1	10
CanadianEMR Rating	9	1	2
Total	58	77	33

## Discussion

The main finding of this study is that vendors, especially of acute care EHRs, provide little product-specific information on their websites. Instead, they try to create favorable attitudes towards EHRs in general, and their products in particular, by other means such as customer testimonials and use of language directed at potential adopters. Obviously, vendor websites are only one source of information about EHRs. Other sources include advertisements in professional journals, salespeople, and peers [2,3,19] . However, the Internet is often the first place people turn to when they seek information about a product [20]. Potential EHR adopters are likely to turn to it at the knowledge stage of the innovation-decision process to become aware of potential offerings and gain basic how-to and principle knowledge. Therefore, the dearth of product specific information on vendor websites could render potential adopters unable to evaluate the various offerings and reach an informed adoption or rejection decision. In particular, the lack of screen captures and demos could make it hard for potential adopters to assess the ease of using the system. Screen captures and demos may also help in forming a mental model of the system [21,22] and thus gaining principle knowledge.

In other domains, many software vendors provide trial versions of their products. Often, fully functional software is free to use for a limited time (eg, SPSS [23]; McAfee Antivirus [24]). In other cases a free “demo” version with limited functionality is provided (eg, RealPlayer [25], Malwarebyte Anti-Malware [26]) [27]. Interestingly, except for OSCAR, which is free, neither of these free trial options was offered by any of the vendors. This may be due to vendors’ reluctance to expose their products to competitors. Additionally, to fully function, EHRs often need to interface with other systems such as laboratory and back-office (eg, billing) [20]. This interoperability may not be possible with free trial versions and, therefore, vendors may prefer live on-site demonstrations over providing a free trial demo. Nevertheless, the lack of trial versions may, to some extent, hinder EHR adoption.

Second, we found that vendors of primary care EHRs provide more information about their products on their websites than vendors of acute care systems. This could be explained by differences in procurement processes. In hospitals, procurement often involves a request for information or proposals (RFI and RFP, respectively) [28]. Vendors may provide detailed information about their products in their responses to RFIs or RFPs and therefore do not feel the need to include it on their websites. In primary care, it is often independent physicians who make purchase decisions, for which they review the various alternatives without going through a formal RFP process [2]. This may also explain why there is often more information on the OntarioMD website than on the individual vendor websites: as physicians must apply for provincial funding through OntarioMD, this would probably be the first place they look for information, and it provides them with a one-stop shop that contains standard information on all certified systems in Ontario. Vendors know that and therefore may not bother with providing complete information on their websites.

### Limitations and Directions for Future Research

One challenge of working with websites is that they are extremely dynamic. Changes to websites may include design modifications, changes to content by the website owner or creator, as well as by others (especially with the advent of Web 2.0), changes to the link structure (both from and to the website), change of location, or removal of the website [29]. Our study captures only a snapshot from 2010, when data were collected. Since then, many sites have been revamped (eg, Eclipsys has been merged into Allscripts; xwave was purchased by Bell and renamed Bell EMR), and some now provide additional information.

All data for this study were taken at face value from the various websites, without accessing the EHRs themselves to verify claims. Gaining access to all systems and producing an impartial comparison would be a valuable information resource. Related to this, substituting the comparison criteria that we used (taken from the IOM) with a different set of criteria, such as technical specifications, would also create a useful information resource that does not currently exist. Additionally, this study included only systems available in Ontario, Canada, and the findings may not apply to other jurisdictions. Similar studies in other jurisdictions could reveal whether our results are indicative of wider trends. Finally, in this study, we looked only at the information presented on vendor websites but not at whether and to what extent it actually affects adoption decisions. It would be interesting to explore the relationships between information on vendor websites and actual EHR adoption levels (eg, market shares). Future research may also look at what other information sources and communication channels are used by physicians and health care organizations in the EHR adoption-decision process, how these resources affect their decisions, and to compare this process with other products and industries (eg, automobile [20]).

### Conclusion

To our knowledge, this study is the first systematic attempt to analyze information presented on EHR vendor websites. Our findings suggest that vendors use various persuasive means to create user confidence and affect their perceptions of EHR systems; however, there is often a lack of specific product information. Greater transparency and provision of concrete product information may benefit both vendors and clients.

## Multimedia Appendix 1

Vendor website analysis data collection form.

## Multimedia Appendix 2

Power Point presentation of home pages of vendor websites included in the analysis. Screen captures taken at the time of data collection (August 2010).

## Multimedia Appendix 3

This Power Point presentation includes screen captures from vendor websites that demonstrate systems' interfaces. Screen captures taken at the time of data collection (August 2010).

## Abbreviations

CHITTA: Canadian Healthcare Information Technology Trade Association
EHR: Electronic Health Record
EMR: Electronic Medical Record
IOM: Institute of Medicine
ITAC: Information Technology Association of Canada
