# Supplementary material for: What Do Electronic Health Record Vendors Reveal About Their Products: An Analysis of Vendor Websites
Source: J Med Internet Res. 2013 Feb 19;15(2):e36. doi: 10.2196/jmir.2312 (PMC3636267; doi:10.2196/jmir.2312)
Supplement: Supplementary file 1 [file jmir_v15i2e36_app1.pdf]

Appendix 1. Vendor website analysis data collection form.

Review Date & Name:

Vendor Name	Vendor Homepage URL	ITAC/ONMD
System Name	System Homepage URL	Primary/Acute Care

Section 1: General Website Analysis (publicly accessible information only)

Date last modified, Data source	Target audience(s) *(eg, public, MDs)*	
Vendor has offices in Canada?<br><br>Where?	Search functionality	Non-English site available?

A. Findability: Description of route from vendor homepage to system homepage *(include multiple options if found)*

B. Navigation *(ease of navigation and backtracking, staying oriented within site)*

C. External credibility *(endorsements/testimonials, client list, HONcode, association logos)*

D. Aesthetics *(eg, readability, consistent visual style and layout, overall design, [language appropriate for audience])*

E. Use of graphical/multimedia features on site; does not have to be related to system *(attach sample with corresponding URLs)*

F. Screenshot of homepage

Section 2: General System Analysis

Date last modified, Data source	Target audience(s) of system *(eg, oncology hospitals)*
Vendor's main/secondary product?	Target audience(s) of information about system

A. Feature list (include languages available, connection to external resources, quotes of "user-friendly interface" etc.)

B. Non-textual description of features

  B1. Screenshots of system interface *(attach corresponding URL)*

  B2. Video/animated system demonstrations

  B3. Use of other graphical/multimedia features to describe system *(attach sample)*

C. Hardware, software, other technical requirements

D. System clients

  D1. Known location of client base *(eg, Ontario, Canada, not stated)*

  D2. Client list/testimonials for this system only *(see also 1C)*

  D3. Customer support services *(contact information, members-only pages, etc.)*

E. Is other information about the system available? *(development history, compliance with standards, etc.)*

F. Screenshot of homepage

Section 3: Multimedia System Features

Quote specific capabilities or attach samples of non-textual information.

Image *(incl. diagnostic images, scanned forms)*	
Audio *(incl. dictation)*	
Video	
Tablet input, handwriting recognition	
Graphing or plotting data	
Other	

Other information:
