# Supplementary material for: What Do Electronic Health Record Vendors Reveal About Their Products: An Analysis of Vendor Websites
Source: J Med Internet Res. 2013 Feb 19;15(2):e36. doi: 10.2196/jmir.2312 (PMC3636267; doi:10.2196/jmir.2312)
Supplement: Supplementary file 3 [file jmir_v15i2e36_app3.ppt]

## Slide 1
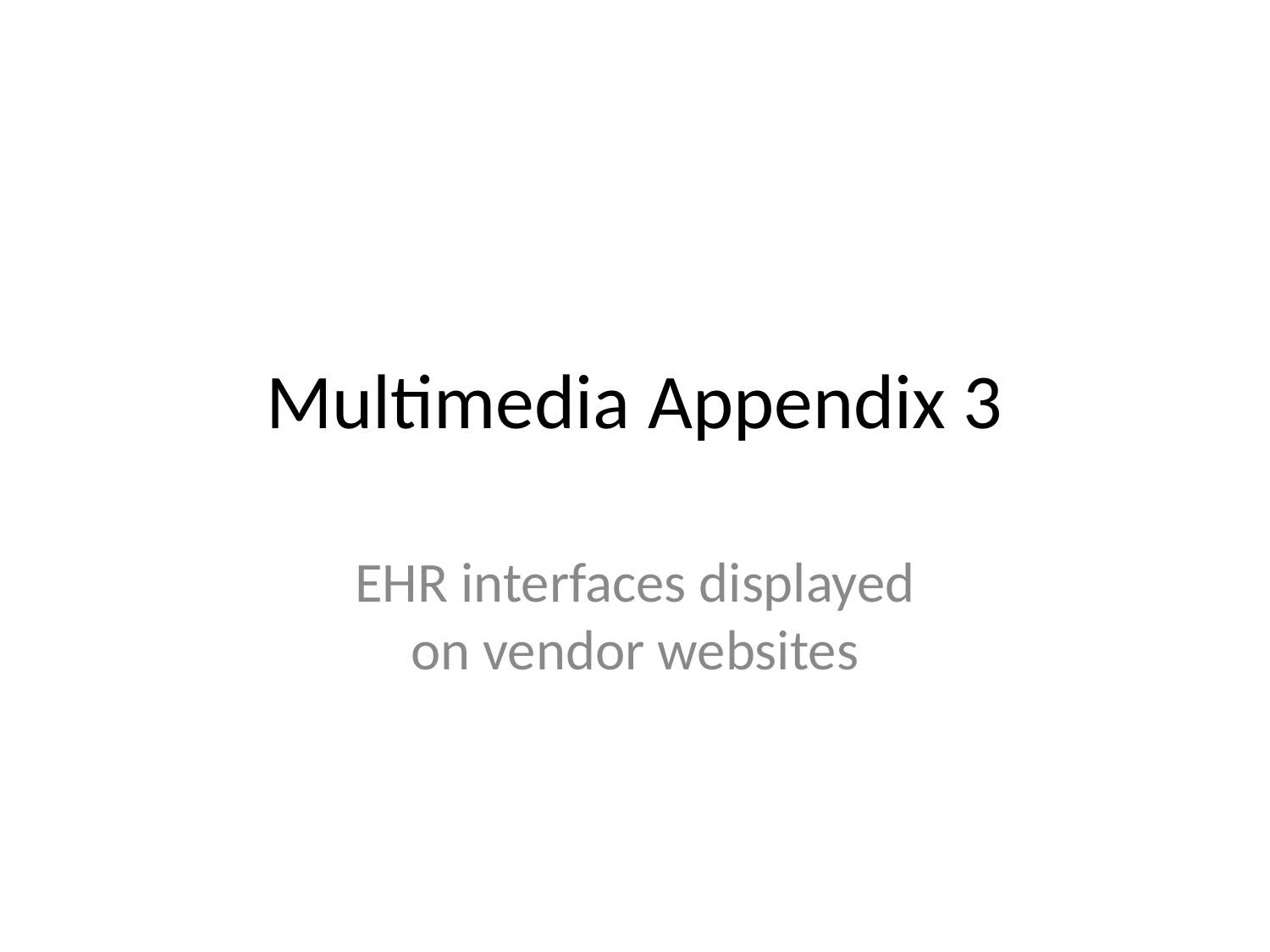

# Multimedia Appendix 3
EHR interfaces displayed on vendor websites

## Slide 2
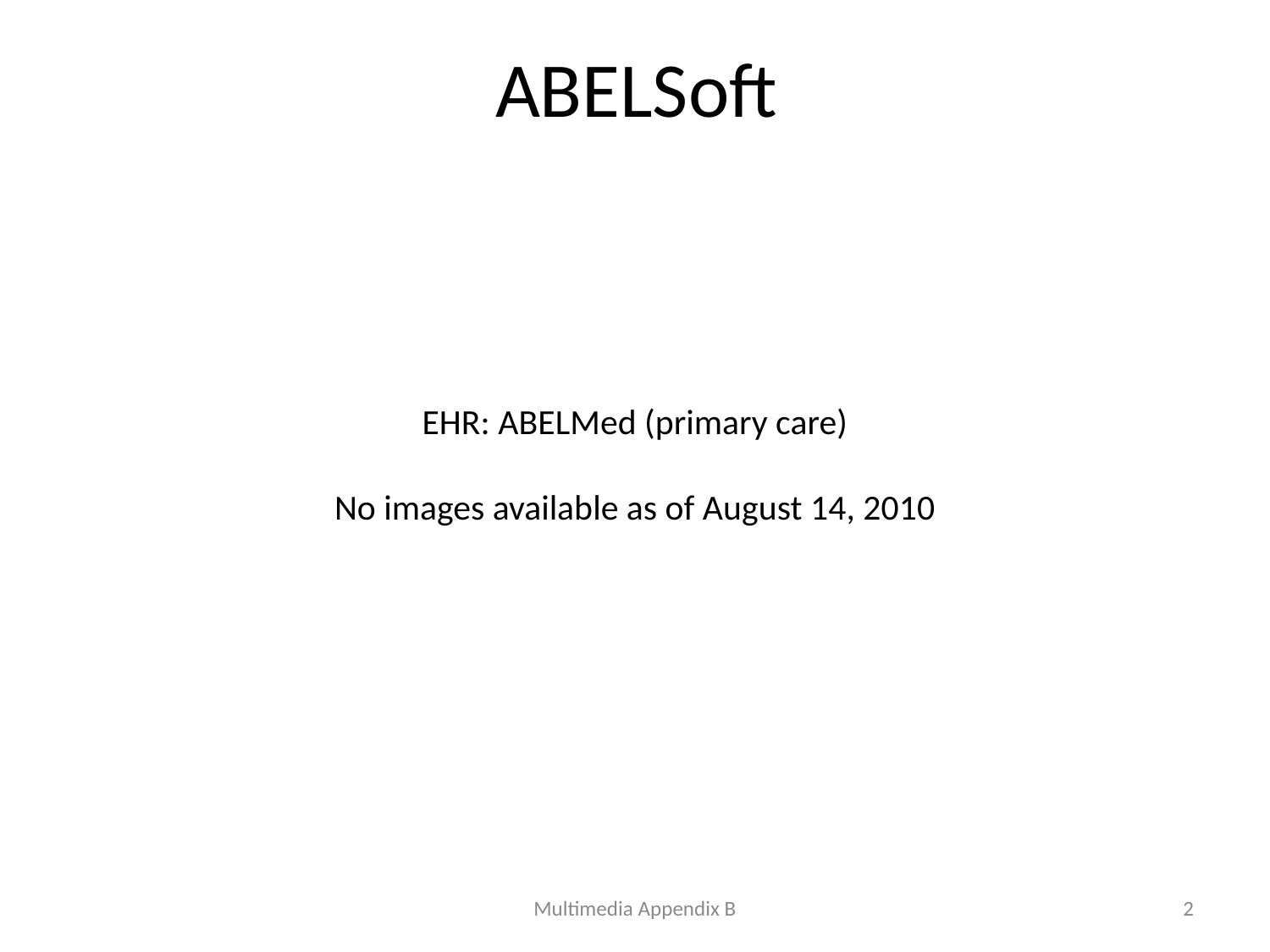

# ABELSoft
EHR: ABELMed (primary care)
No images available as of August 14, 2010
Multimedia Appendix B
<number>

## Slide 3
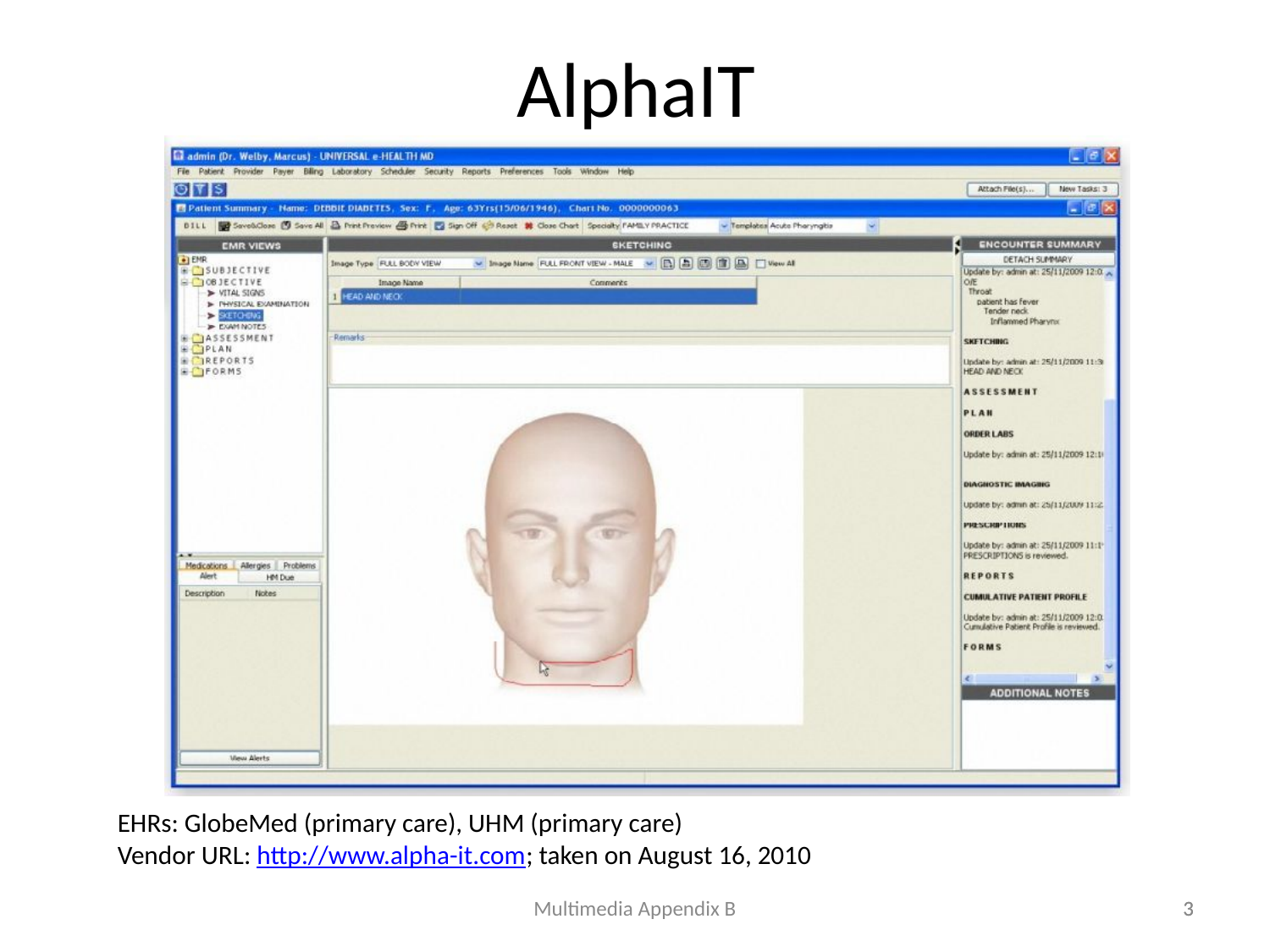

AlphaIT
EHRs: GlobeMed (primary care), UHM (primary care)
Vendor URL: http://www.alpha-it.com; taken on August 16, 2010
Multimedia Appendix B
<number>
<number>

## Slide 4
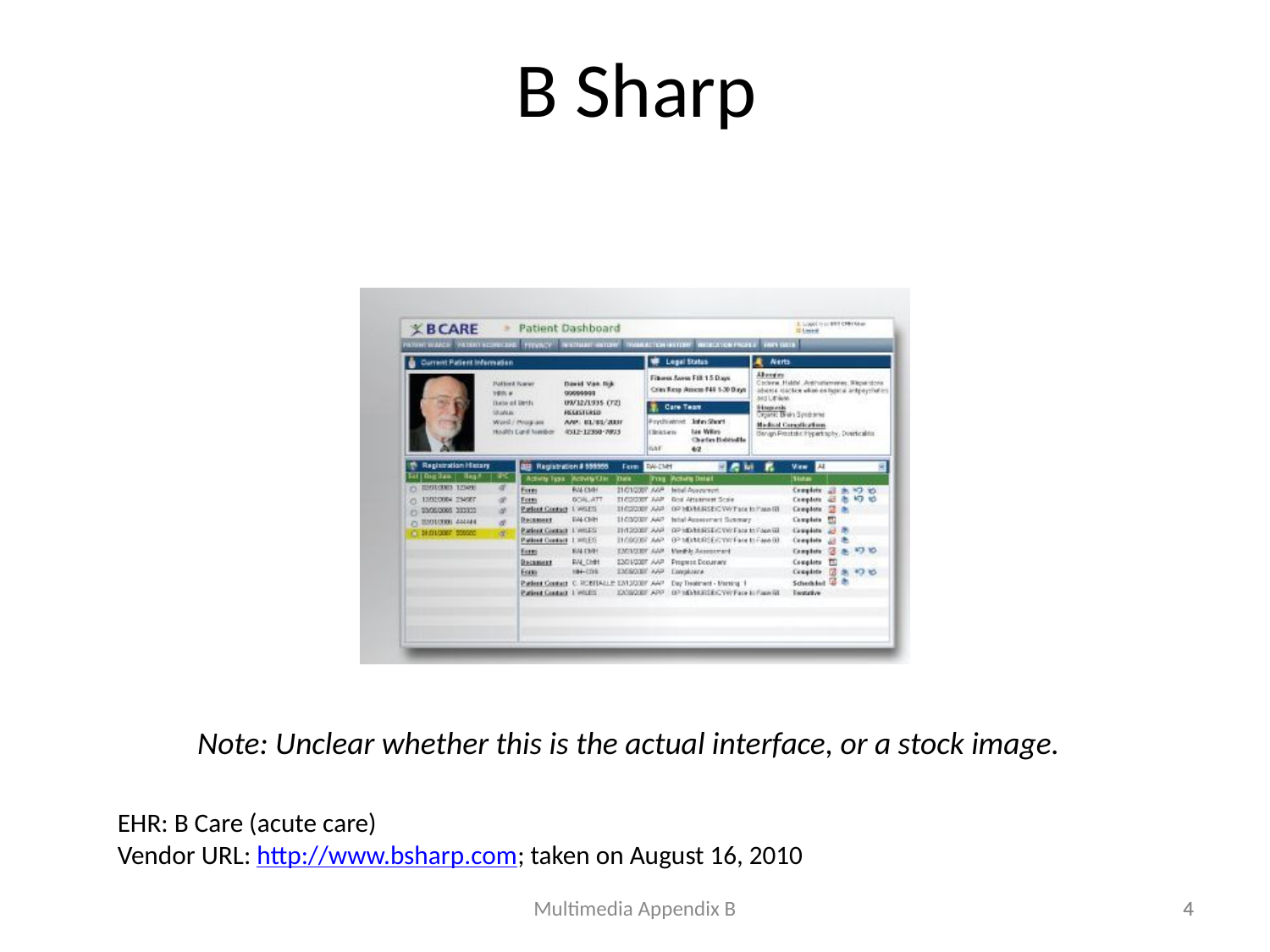

# B Sharp
Note: Unclear whether this is the actual interface, or a stock image.
EHR: B Care (acute care)
Vendor URL: http://www.bsharp.com; taken on August 16, 2010
Multimedia Appendix B
<number>
<number>

## Slide 5
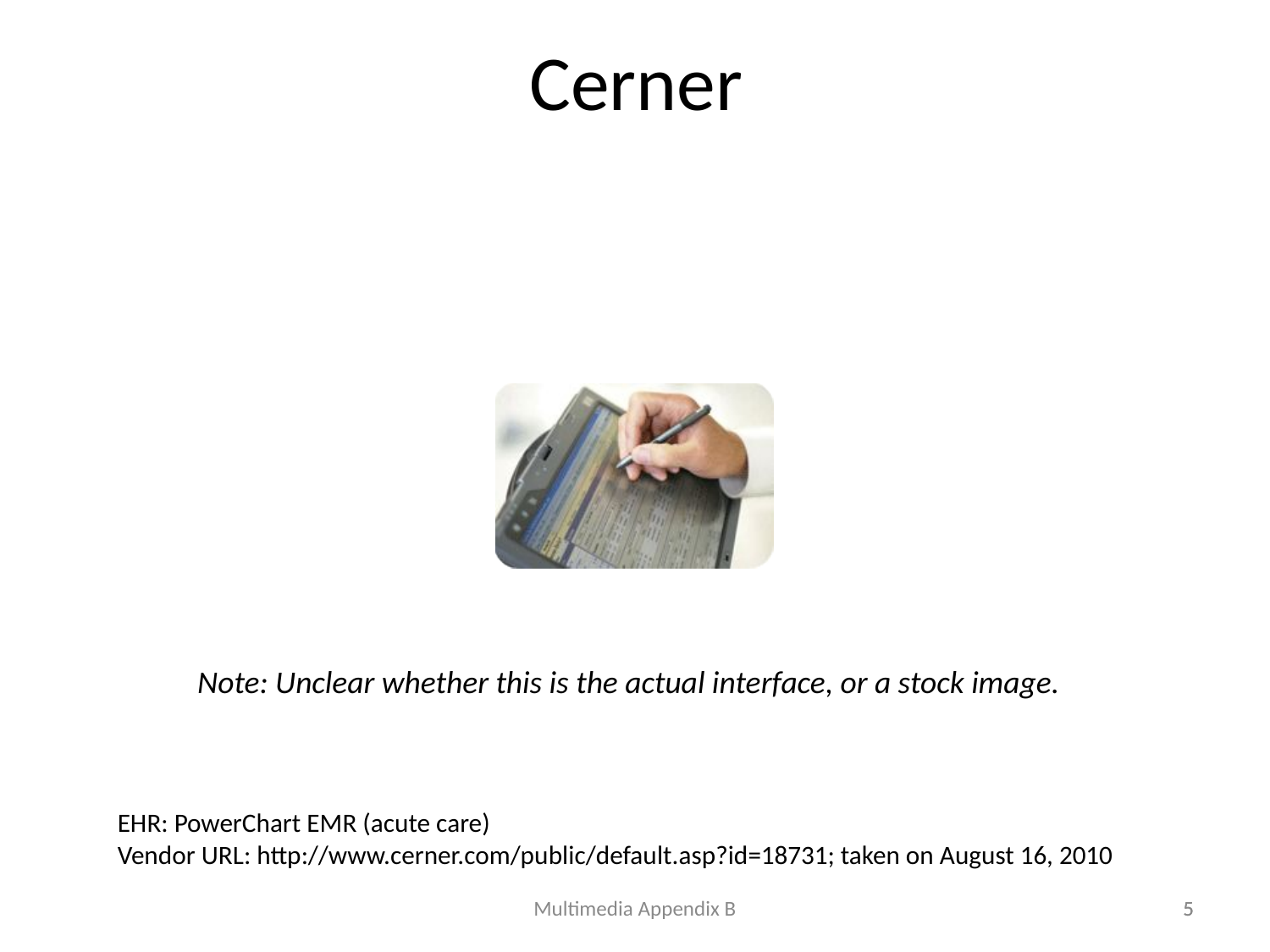

Cerner
Note: Unclear whether this is the actual interface, or a stock image.
EHR: PowerChart EMR (acute care)
Vendor URL: http://www.cerner.com/public/default.asp?id=18731; taken on August 16, 2010
Multimedia Appendix B
<number>
<number>

## Slide 6
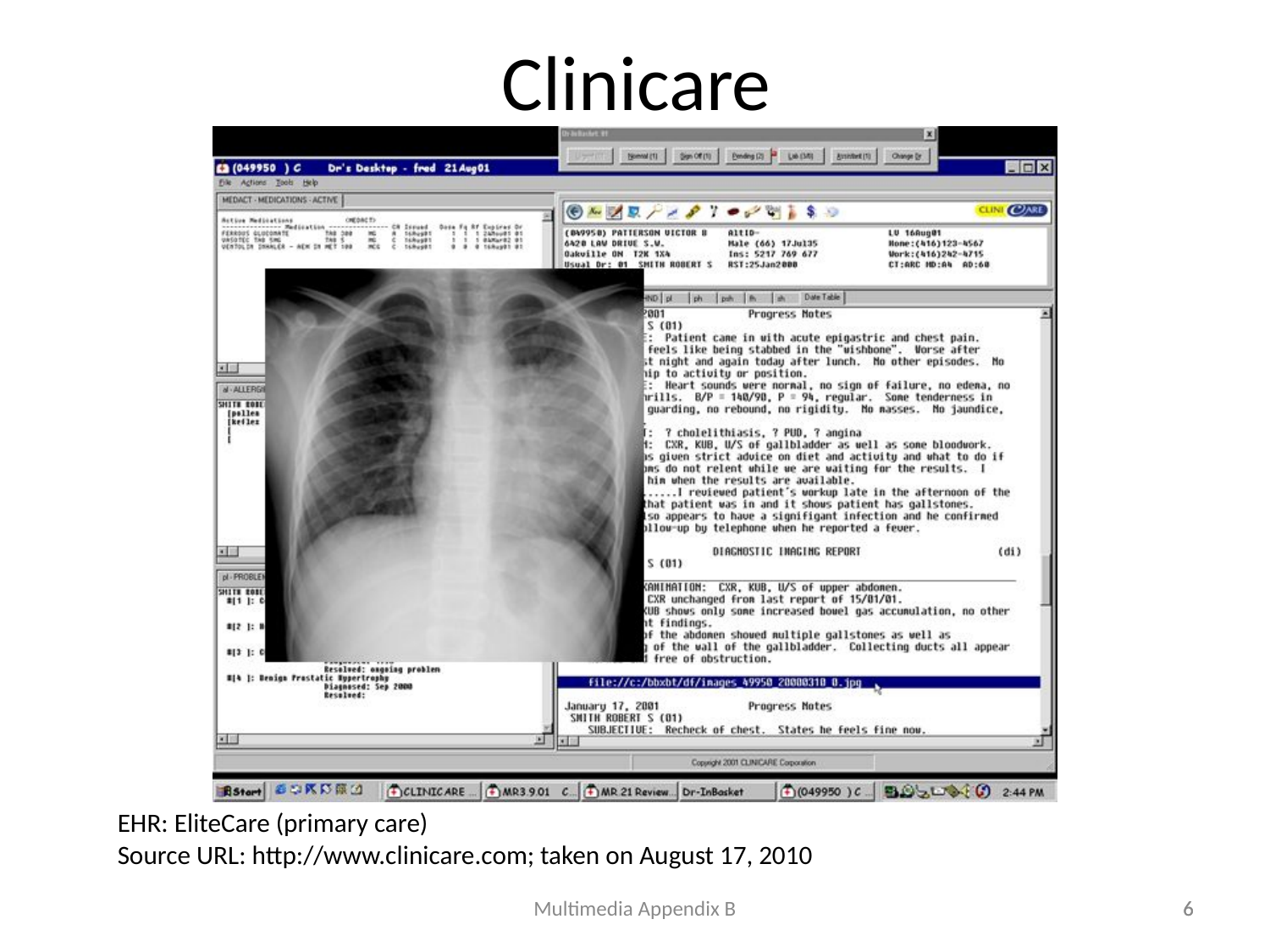

Clinicare
EHR: EliteCare (primary care)
Source URL: http://www.clinicare.com; taken on August 17, 2010
Multimedia Appendix B
<number>
<number>

## Slide 7
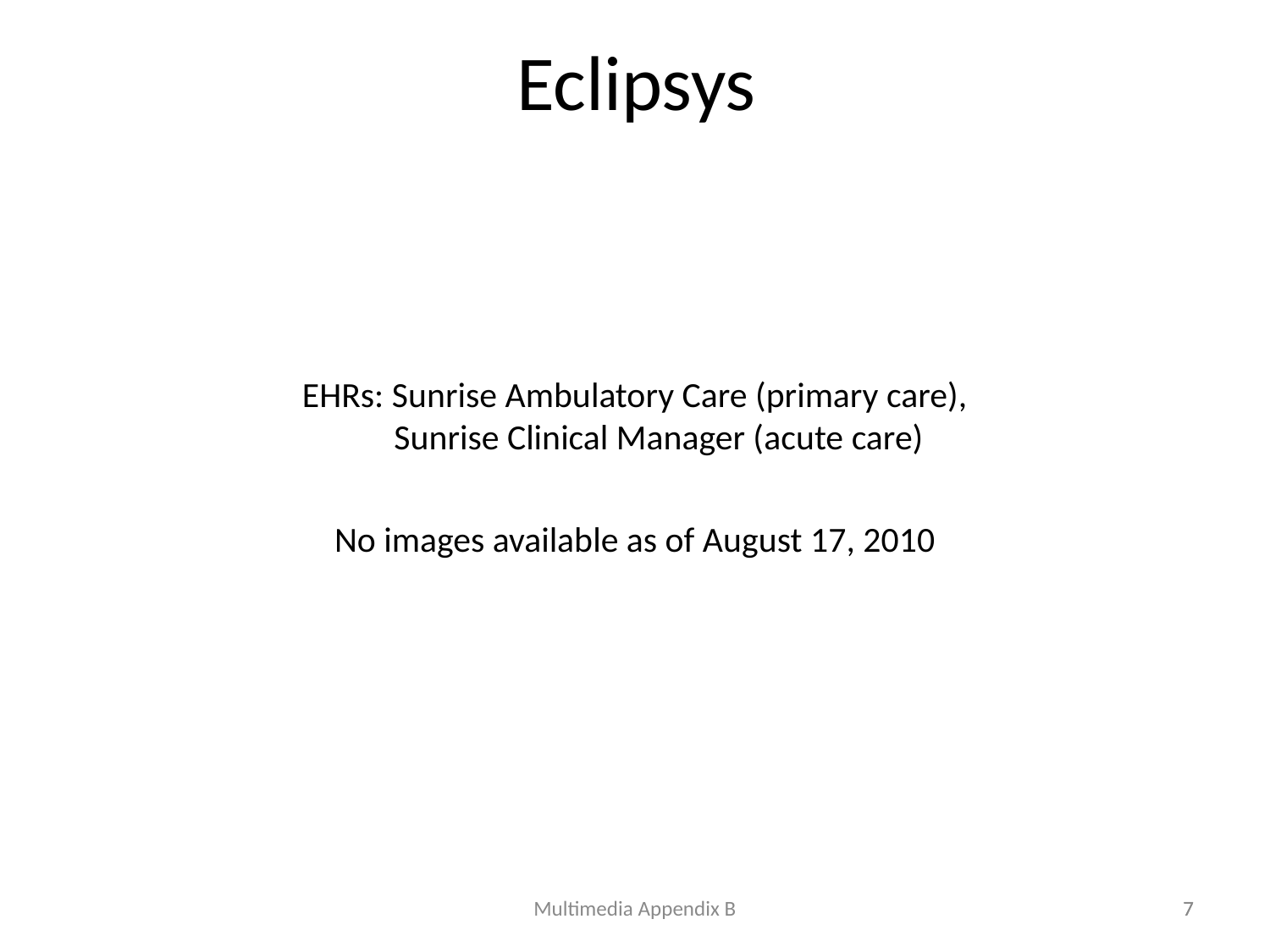

Eclipsys
EHRs: Sunrise Ambulatory Care (primary care), Sunrise Clinical Manager (acute care)
No images available as of August 17, 2010
Multimedia Appendix B
<number>
<number>

## Slide 8
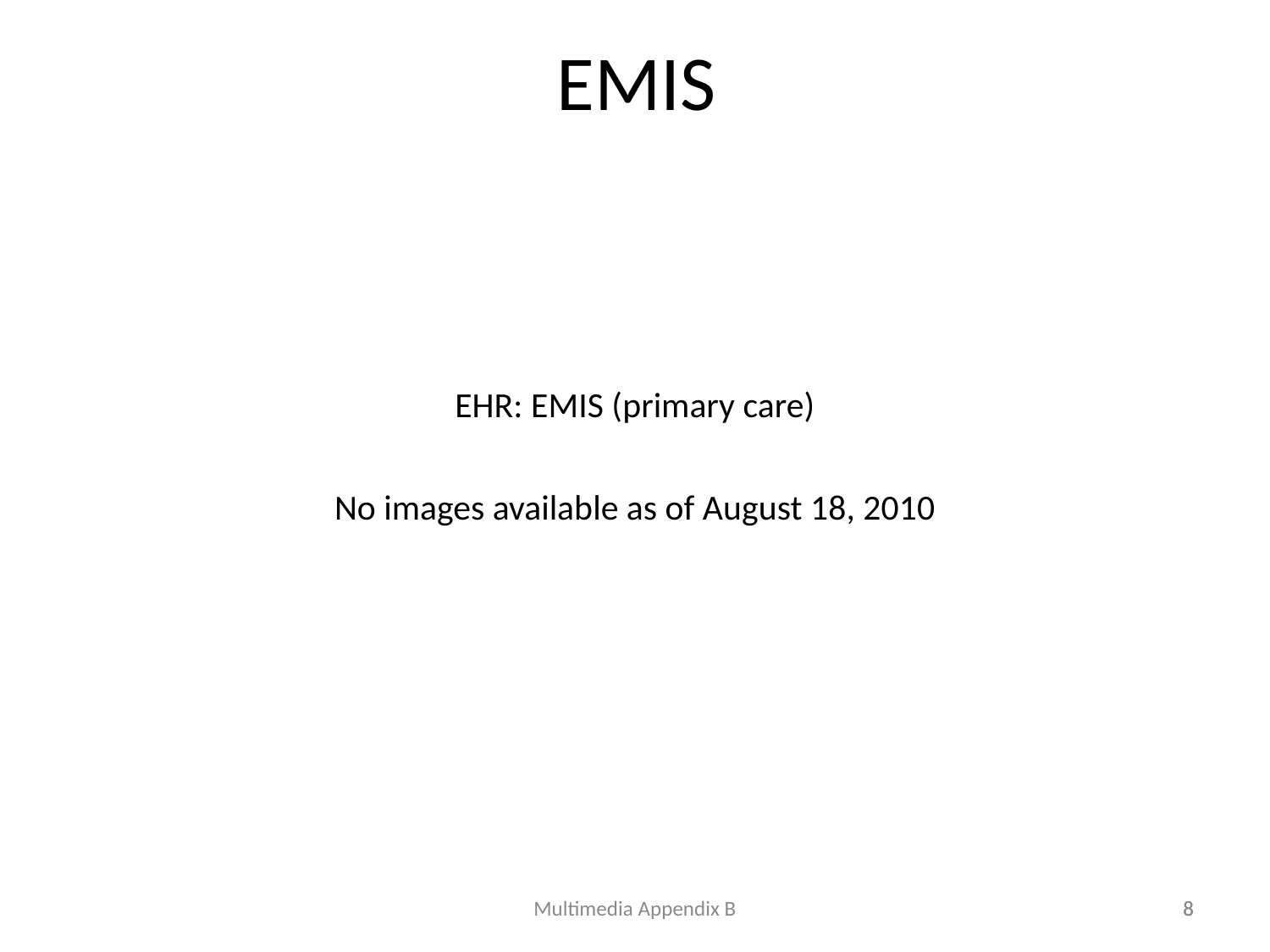

EMIS
EHR: EMIS (primary care)
No images available as of August 18, 2010
Multimedia Appendix B
<number>
<number>

## Slide 9
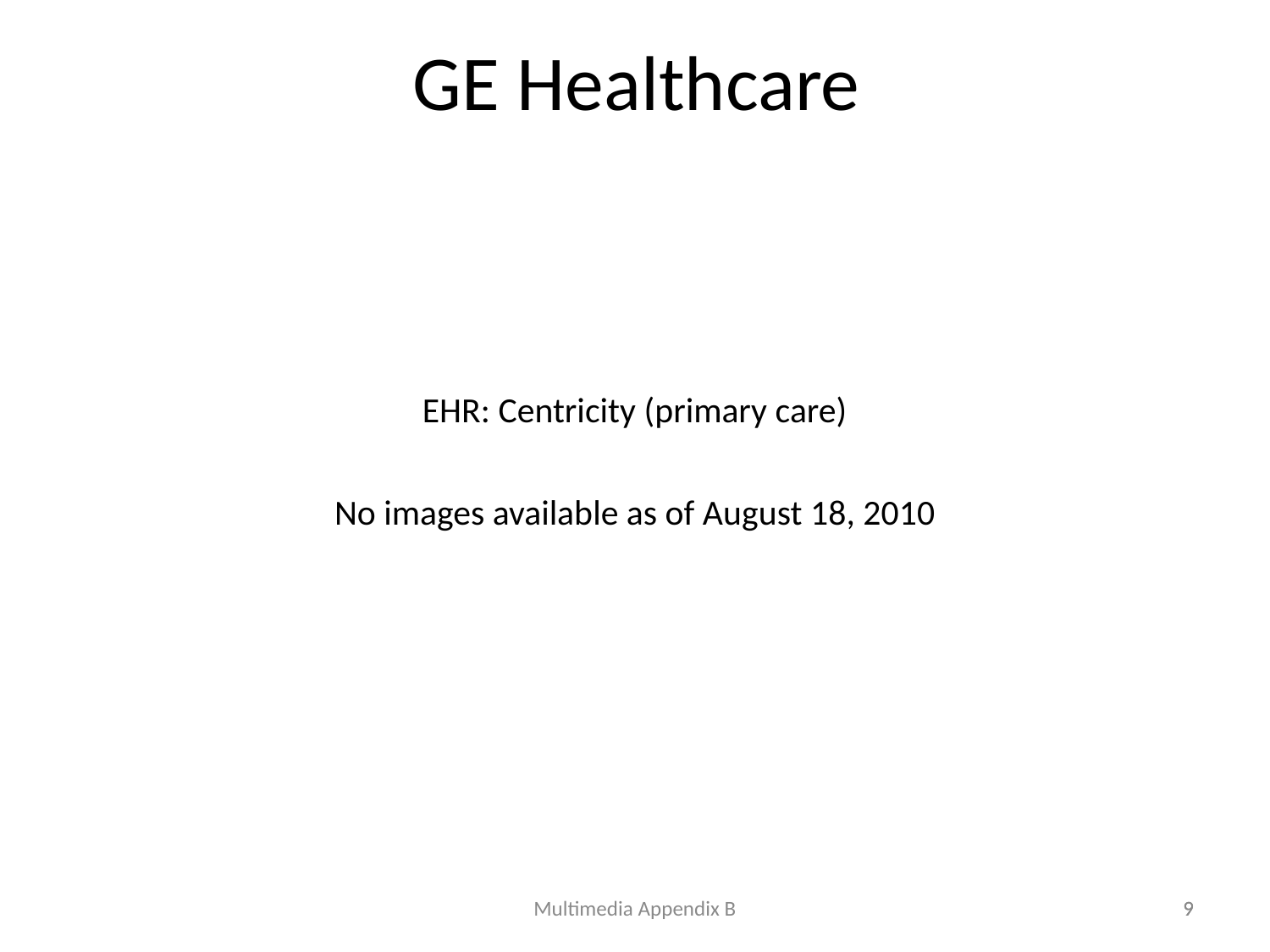

GE Healthcare
EHR: Centricity (primary care)
No images available as of August 18, 2010
Multimedia Appendix B
<number>
<number>

## Slide 10
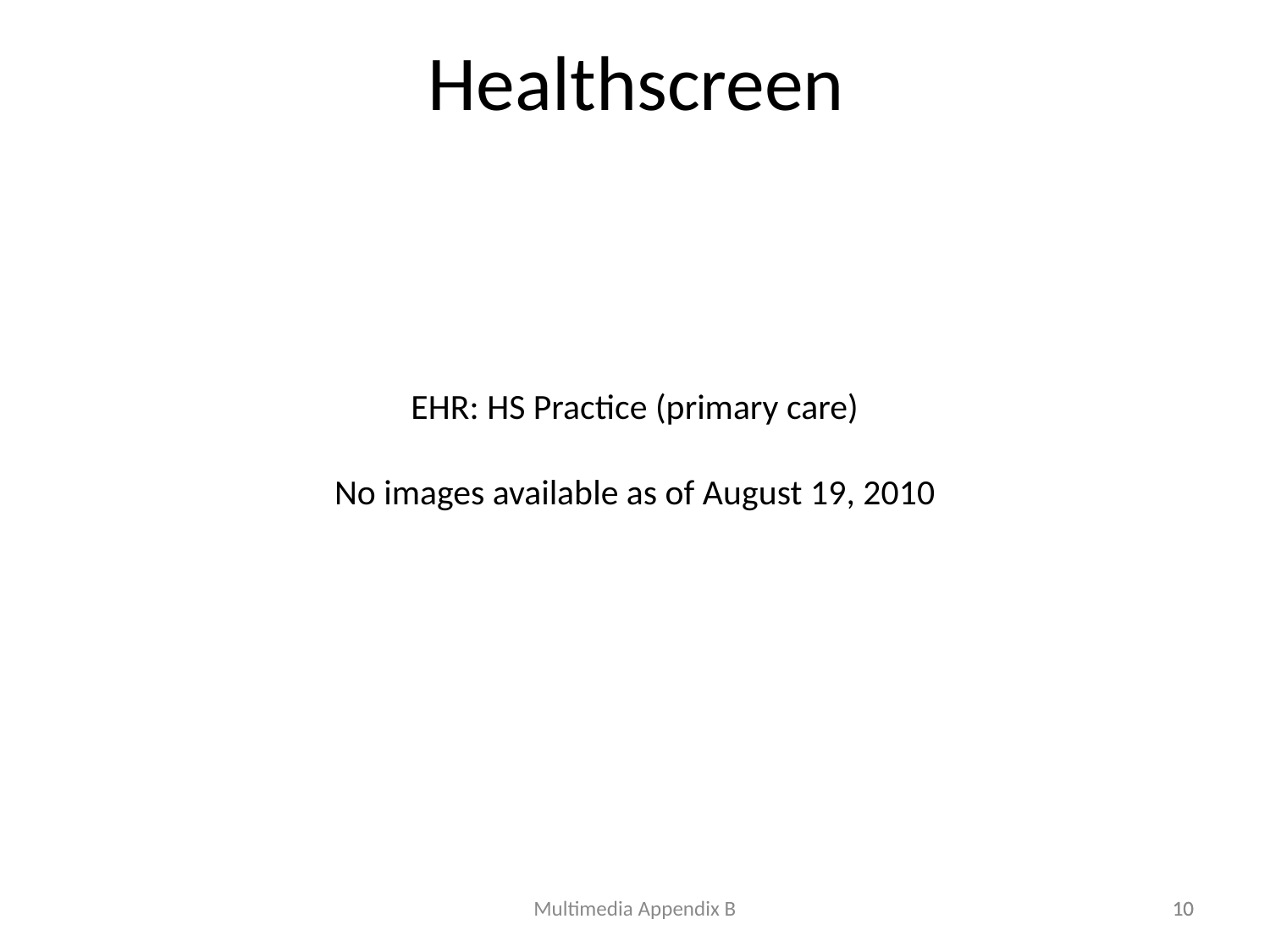

Healthscreen
EHR: HS Practice (primary care)
No images available as of August 19, 2010
Multimedia Appendix B
<number>
<number>

## Slide 11
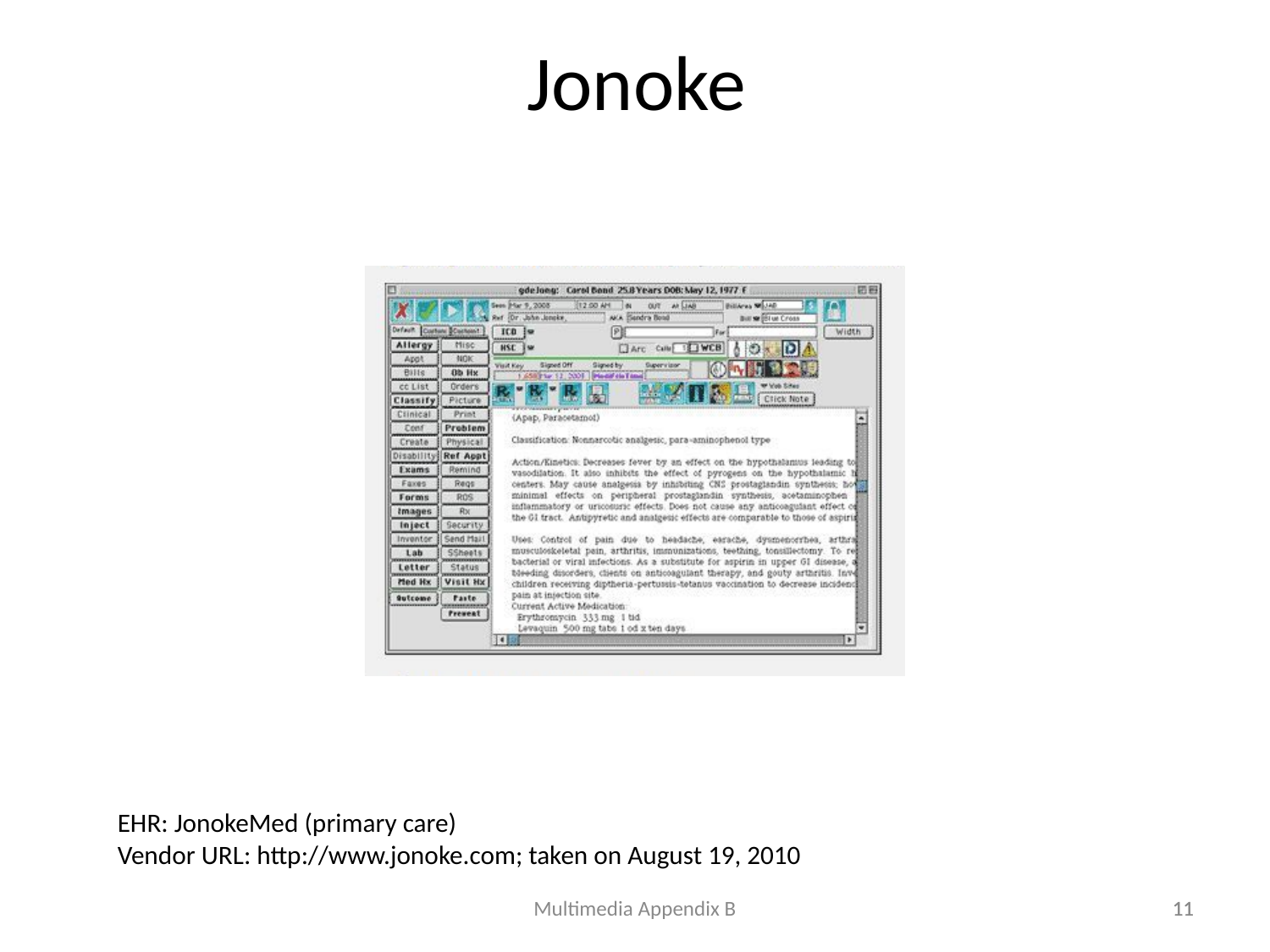

Jonoke
EHR: JonokeMed (primary care)
Vendor URL: http://www.jonoke.com; taken on August 19, 2010
Multimedia Appendix B
<number>
<number>

## Slide 12
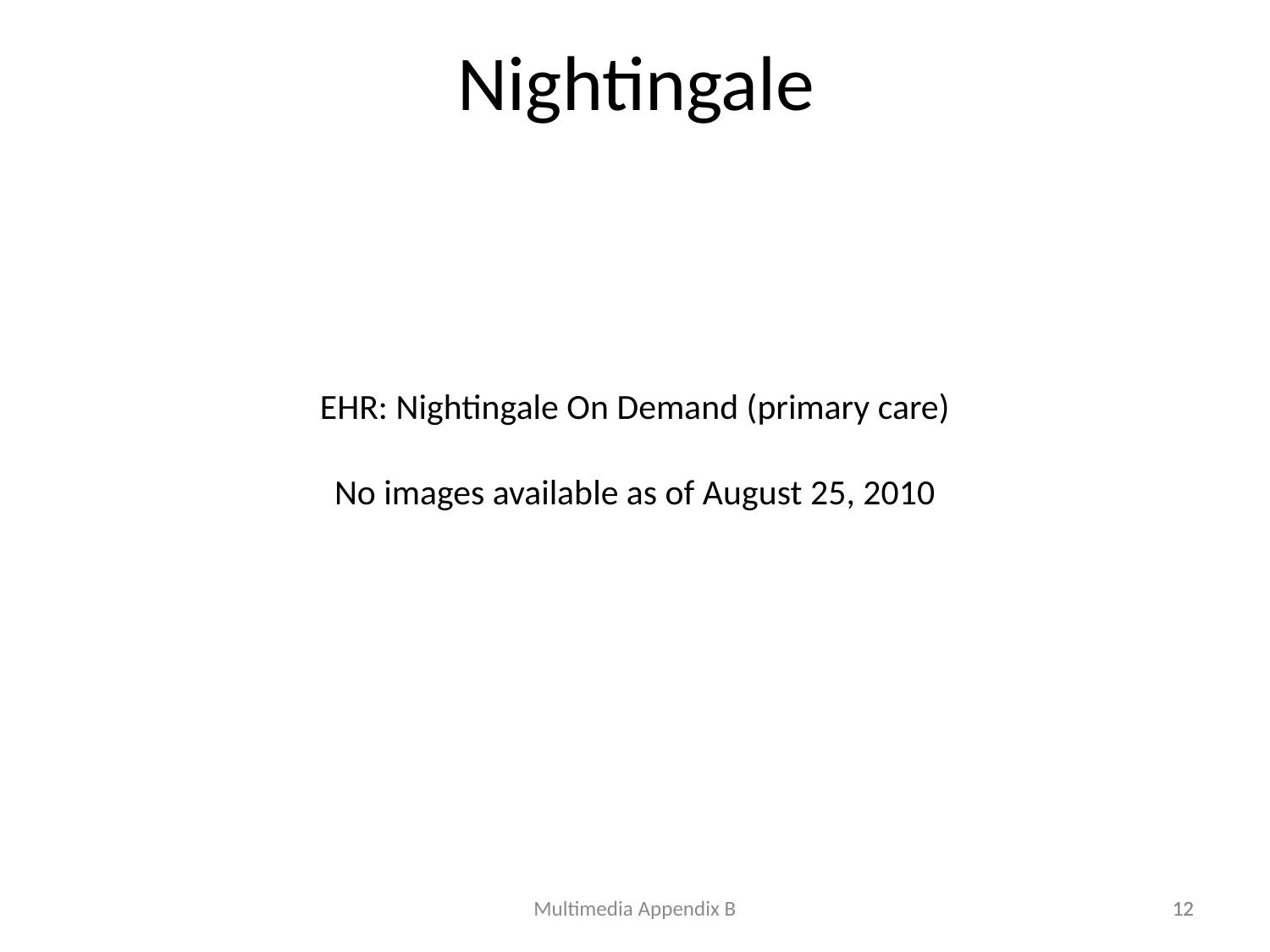

Nightingale
EHR: Nightingale On Demand (primary care)
No images available as of August 25, 2010
Multimedia Appendix B
<number>
<number>

## Slide 13
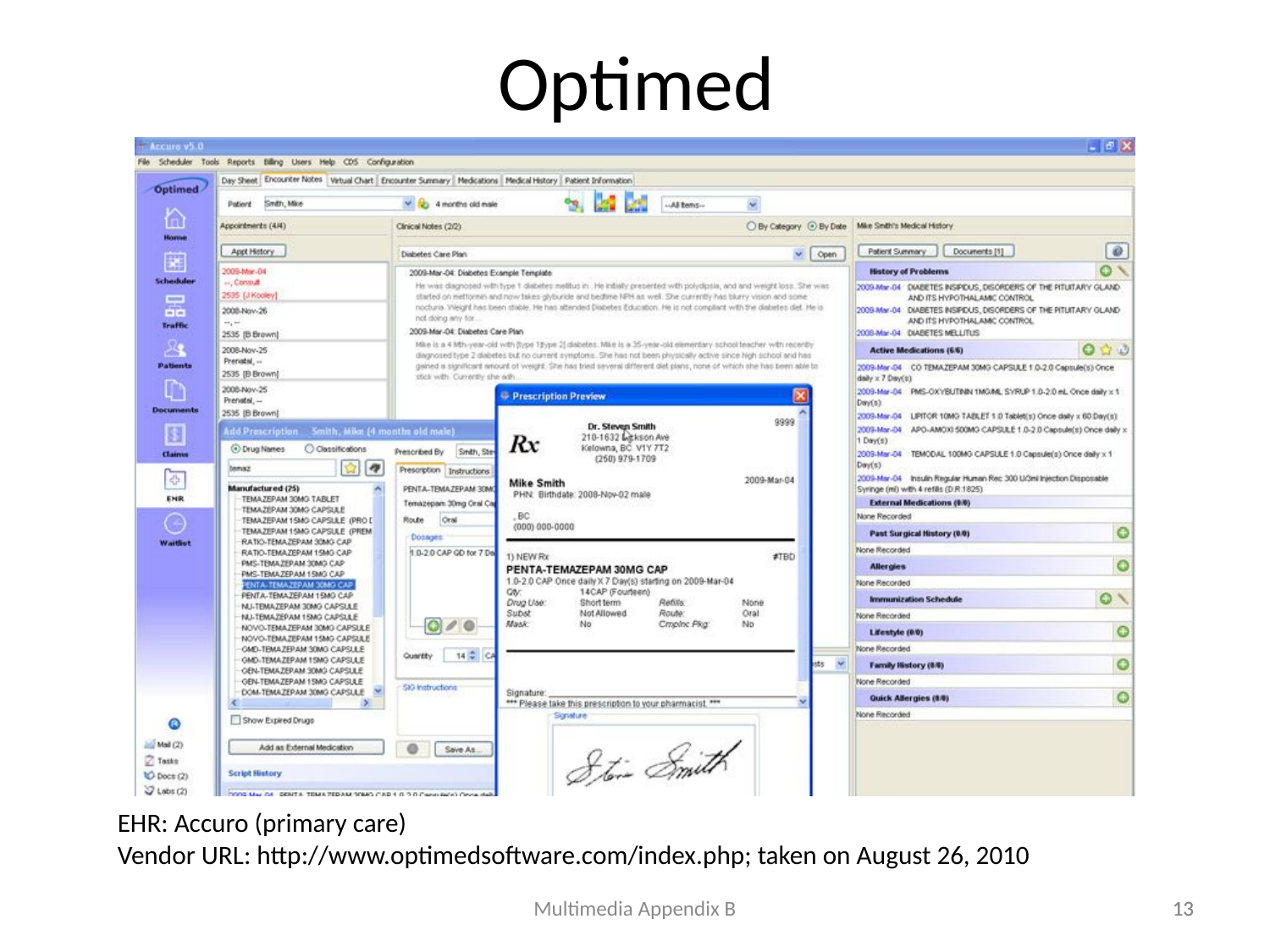

Optimed
EHR: Accuro (primary care)
Vendor URL: http://www.optimedsoftware.com/index.php; taken on August 26, 2010
Multimedia Appendix B
<number>
<number>

## Slide 14
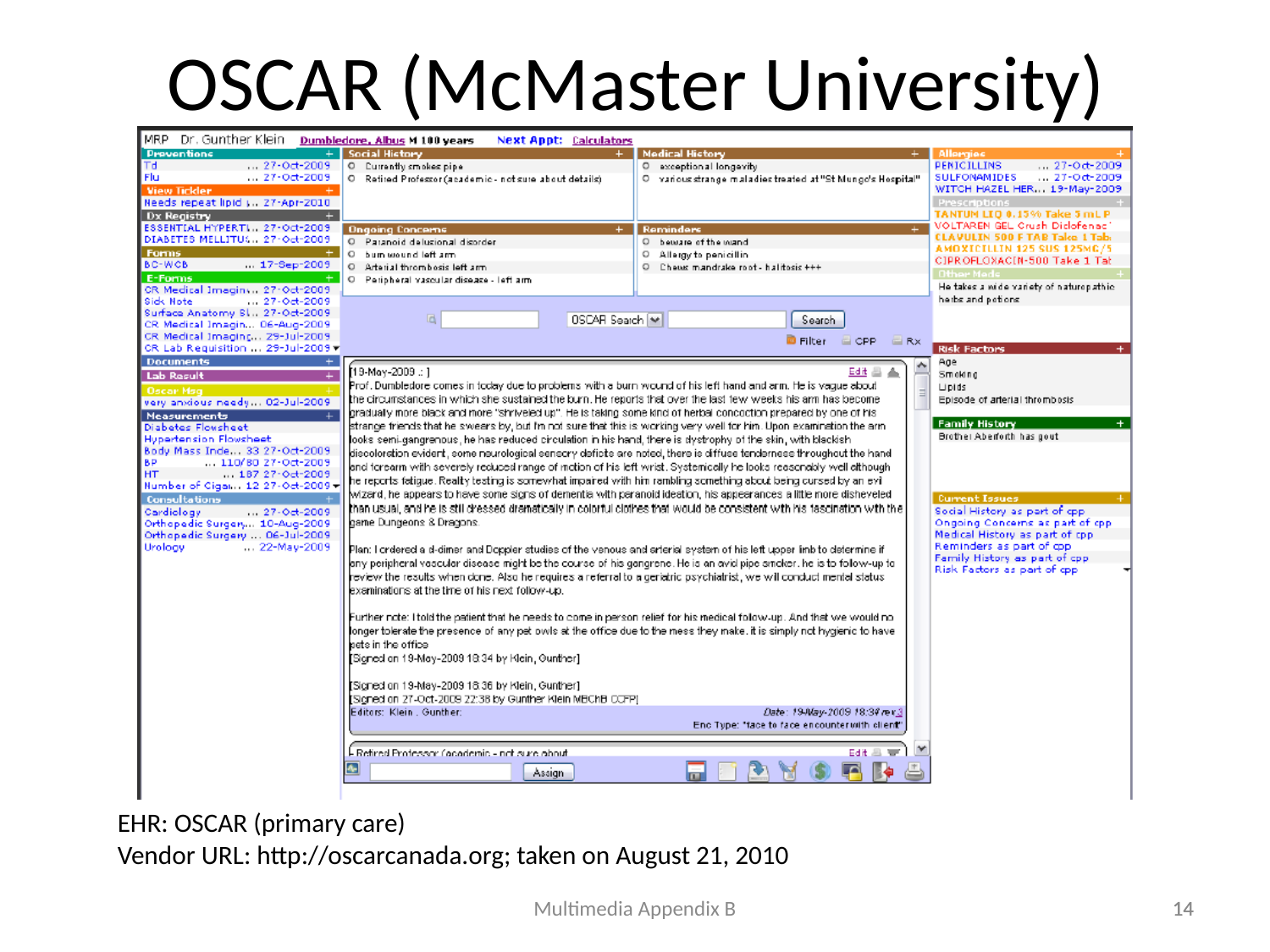

OSCAR (McMaster University)
EHR: OSCAR (primary care)
Vendor URL: http://oscarcanada.org; taken on August 21, 2010
Multimedia Appendix B
<number>
<number>

## Slide 15
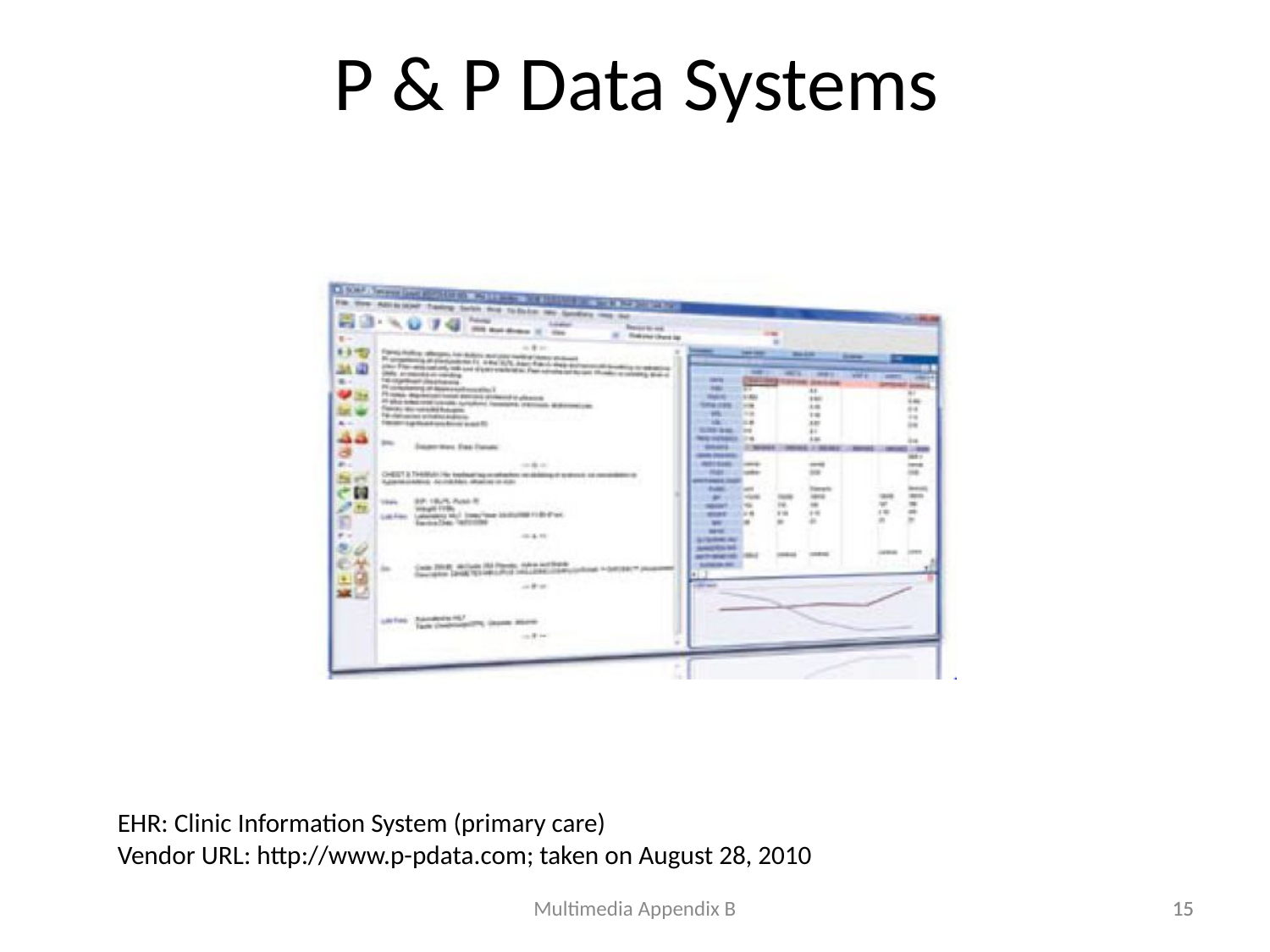

P & P Data Systems
EHR: Clinic Information System (primary care)
Vendor URL: http://www.p-pdata.com; taken on August 28, 2010
Multimedia Appendix B
<number>
<number>

## Slide 16
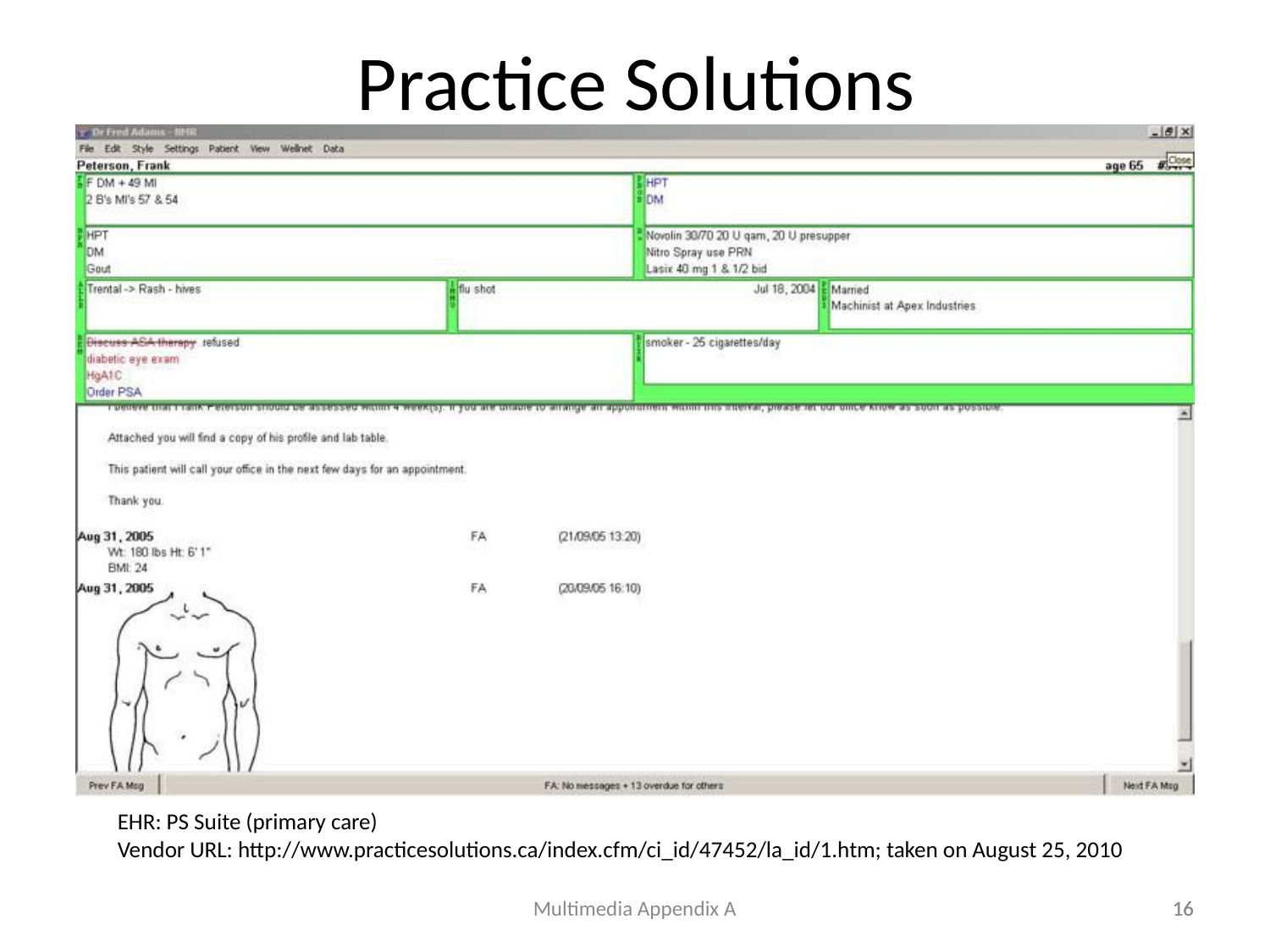

Practice Solutions
EHR: PS Suite (primary care)
Vendor URL: http://www.practicesolutions.ca/index.cfm/ci_id/47452/la_id/1.htm; taken on August 25, 2010
Multimedia Appendix A
<number>
<number>

## Slide 17
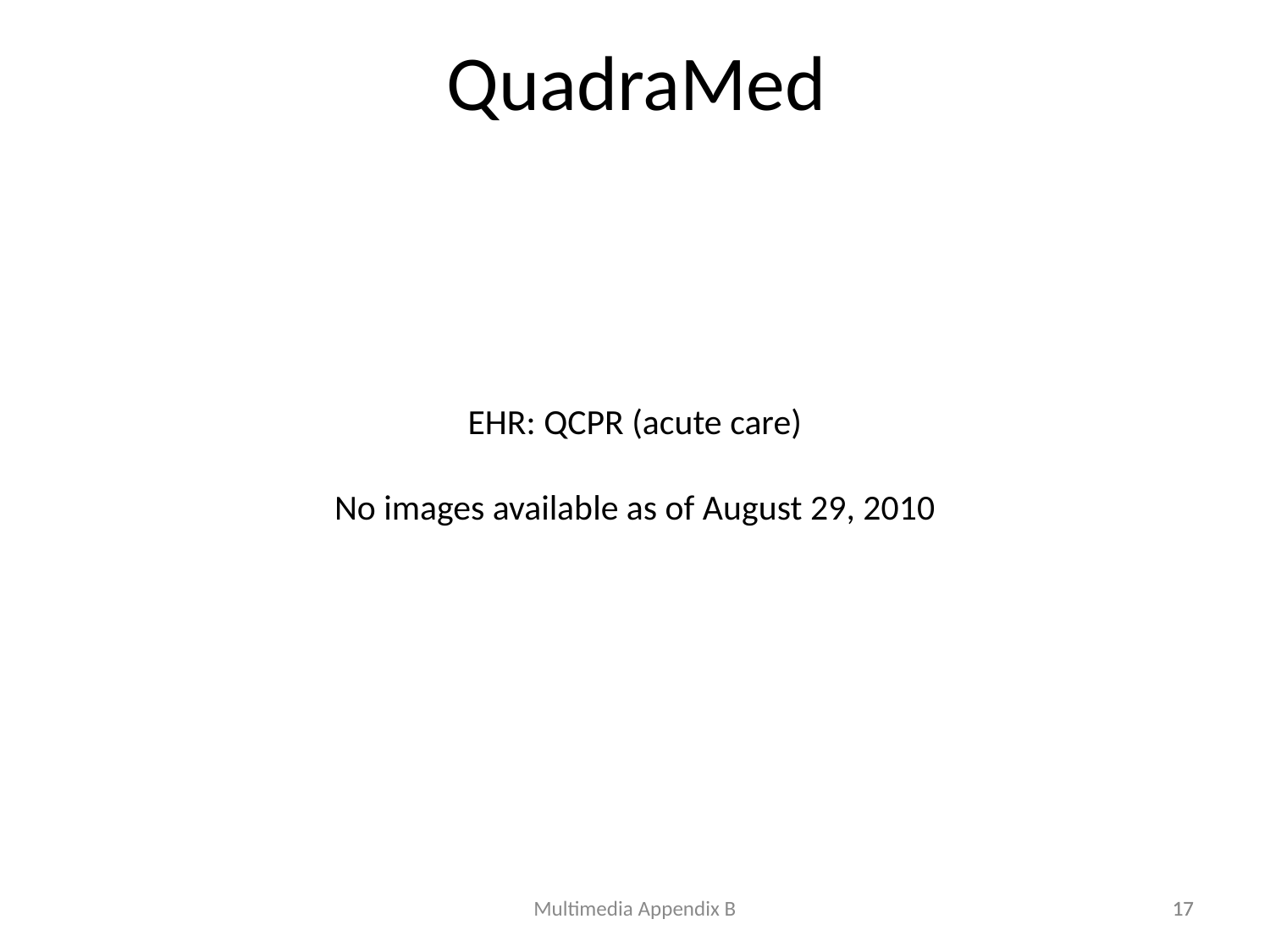

QuadraMed
EHR: QCPR (acute care)
No images available as of August 29, 2010
Multimedia Appendix B
<number>
<number>

## Slide 18
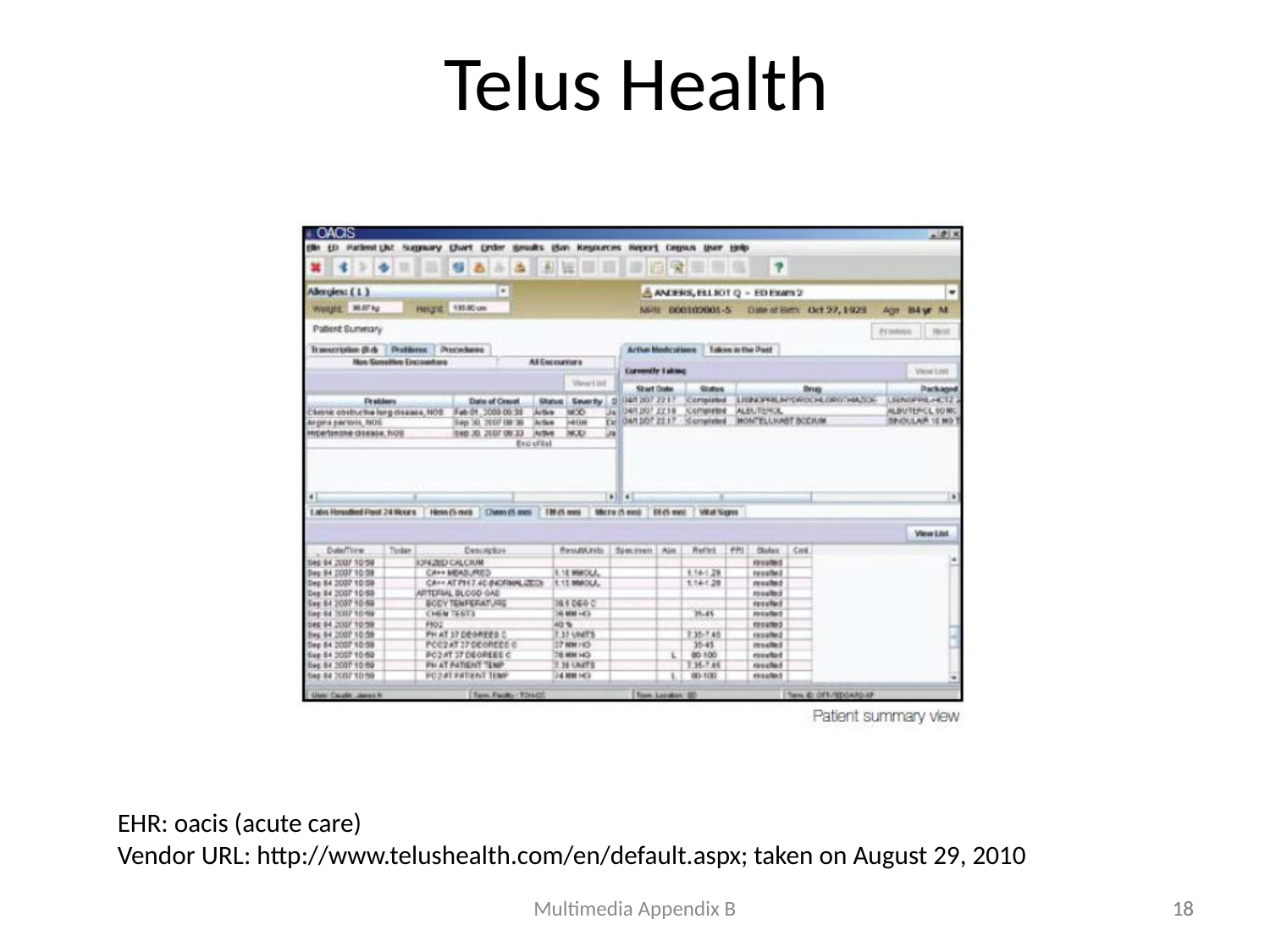

Telus Health
EHR: oacis (acute care)
Vendor URL: http://www.telushealth.com/en/default.aspx; taken on August 29, 2010
Multimedia Appendix B
<number>
<number>

## Slide 19
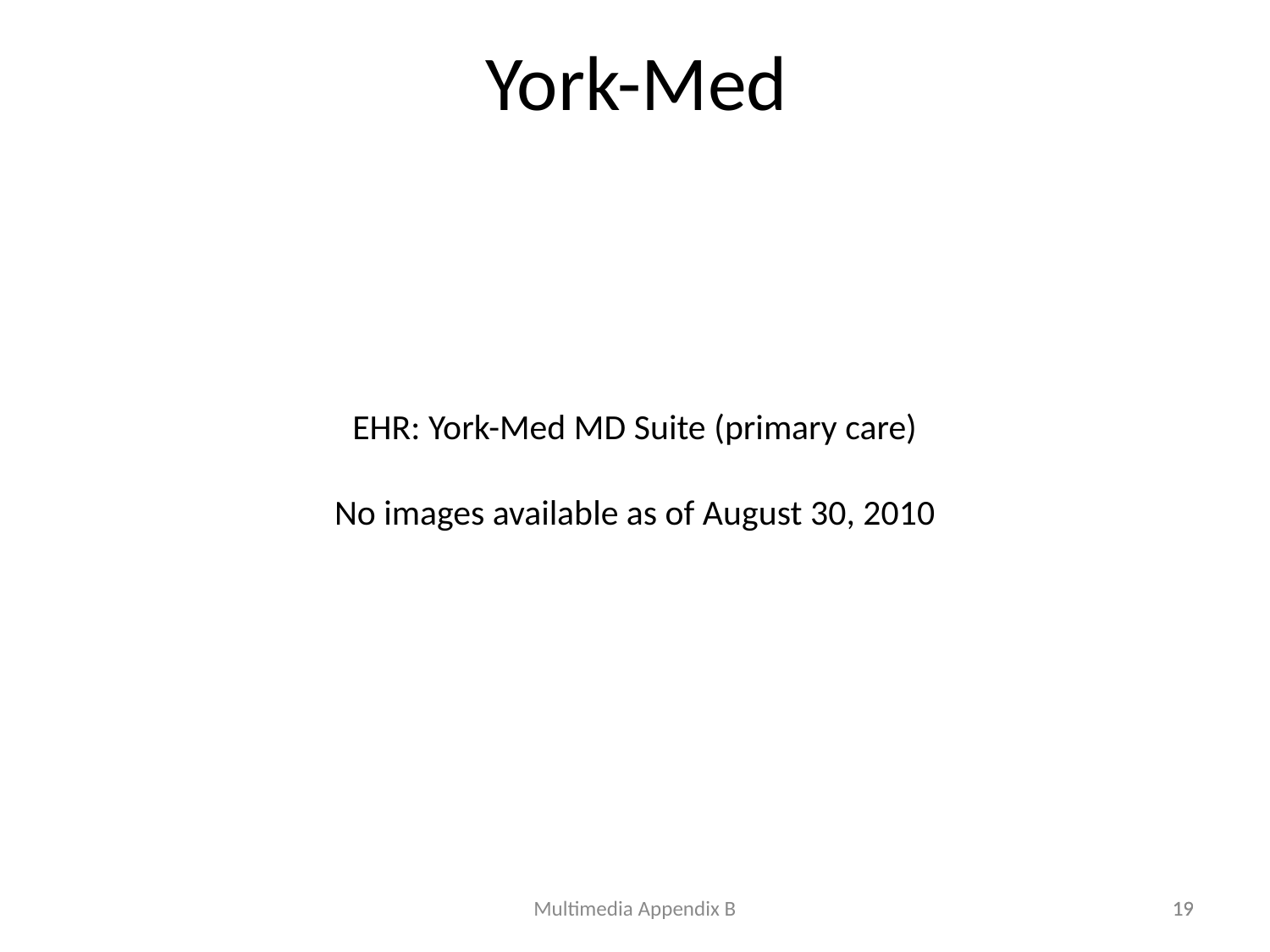

York-Med
EHR: York-Med MD Suite (primary care)
No images available as of August 30, 2010
Multimedia Appendix B
<number>
<number>
